# Neuro-immune interactions in urticaria:a pruritus-centric dissection

**DOI:** 10.3389/fimmu.2026.1782901

**Published:** 2026-04-02

**Authors:** Chunxi Ke, Ni Ma, Gang Chen, Yuxu Yao, Jiang Ji, Qingqing Jiao

**Affiliations:** 1 Department of Dermatology, The Second Affiliated Hospital of Soochow University, Suzhou, China; 2 Department of Neurosurgery and Brain and Nerve Research Laboratory, The First Affiliated Hospital of Soochow University, Suzhou, China; 3 Department of Dermatology, Affiliated Women’s Hospital of Jiangnan University, Wuxi, China; 4 Central Research Laboratory, The First Affiliated Hospital of Soochow University, Suzhou, China; 5 Department of Dermatology, The First Affiliated Hospital of Soochow University, Suzhou, China

**Keywords:** itch, neuroimmune interactions, neuropeptide, therapeutic targets, urticaria

## Abstract

Urticaria is a mast cell-driven skin disease, characterized by itchiness and transient wheal development. Although histamine released from activated mast cells is central to disease pathogenesis, increasing clinical evidence indicates that a subset of patients exhibit limited efficacy to antihistamines and biologics such as omalizumab. This therapeutic limitation emphasizes the involvement of additional, non-histaminergic pathways in disease persistence. Recent studies highlight the pivotal role of neuroimmune interactions, the crosstalk between the immune and nervous systems, especially in modulating type 2 inflammation and itch. In urticaria, neuroimmune mechanisms amplify pruritic signaling, and promote neurogenic inflammation, and sustain mast cell activation, collectively contributing to chronicity and treatment resistance. Deciphering these neuroimmune loops provides new insight into urticaria pathophysiology and identifies potential molecular targets for therapy. A growing number of biologics targeting neuroimmune pathways are showing encouraging efficacy in early clinical trials. This review adopts a pruritus-centered perspective to synthesize updated advances in neuroimmune research related to urticaria and to outline future directions for mechanism-based therapy.

## 1 Introduction

Urticaria is an inflammatory dermatosis driven by mast cells, presenting as itchy wheals and/or angioedema. It is categorized based on duration as acute (<6 weeks) or chronic (≥6 weeks) and further classified by triggers as spontaneous (unpredictable flares) or inducible (elicited by physical or contact stimuli) (1). Chronic spontaneous urticaria (CSU) is the predominant chronic form, globally prevalent with a prolonged clinical course. Challenges in current management include: (i) a subset of patients (11–14%) experiencing disease activity for over five years (2–4), incurring significant healthcare costs comparable to hypertension and diabetes (5), yet validated biomarkers capable of forecasting disease duration remain absent; (ii) the prevailing “autoimmune-Immunoglobulin E (IgE)/autoantibody–mast cell–histamine” paradigm fails to account for therapeutic failure in ≥25% of subjects receiving high-dose second-generation H1-antihistamines and incomplete response in ~32% of omalizumab-treated individuals (6, 7); and (iii) the synchronous, waxing–waning recurrence of wheal, oedema and pruritus implies the existence of amplification circuits that lie beyond the reach of canonical immune pathway.

The skin serves as a complex interface involving the nervous, immune, and endocrine systems (8). Within this interface, sensory neurons in the skin detect allergens, cytokines, and immune molecules, transmit itch-related signals, and release neuropeptides and neurotransmitters that enhance local inflammation (9, 10). This process establishes a self-reinforcing cycle of itching and inflammation. Studies have shown increased levels of neuropeptides in both lesional skin tissue and plasma of CSU individuals, while psychophysiological stress further augments this circuitry. Initial clinical trials focusing on neuro-immune interactions, such as vixarelimab (targeting Interleukin-31 (IL-31) through Oncostatin M Receptor β (OSMRβ)) and dupilumab (targeting Interleukin-4 Receptor (IL-4Rα)), have shown promising early results. However, the precise spatiotemporal dynamics and regulatory mechanisms of neuro-immune communication remain poorly understood, posing a significant challenge to achieving comprehensive disease management.

Given that pruritus is the cardinal and most distressing symptom that precipitates medical consultation and exhibits temporal concordance with inflammatory manifestations (wheal and oedema), the present reviews focus on pruritus as the central aspect of analysis. We comprehensively summarize existing insights on neuro-immune interactions in urticaria, delineate key molecular mediators and signaling axes. This summary aims to lay a theoretical foundation for precise drug utilization and coordinated intervention strategy aimed at neuroimmune nodes.

## 2 Pruritus in urticaria

### 2.1 Characteristic features of pruritus

Urticarial pruritus is characterized by transient yet recurring sensations of burning and stinging that often intensify at night and in response to emotional stress, heat, sweating, or xerosis (11, 12). Unlike most chronic pruritic dermatoses, urticarial lesions seldom result in excoriations or nodular prurigo (13). Patients typically alleviate discomfort through friction, tapping, or cold compresses rather than scratching, indicating a predominantly neurogenic rather than eczematous itch phenotype. Quantitative sensory profiling has demonstrated a strong positive correlation between perceived itch intensity and subjective “heat” ratings (14). Episodes of pruritus coincide with the appearance of wheals, and both resolve spontaneously within minutes to hours, exhibiting a rhythmic “wave-like” pattern that parallels the cyclic process of mast cell degranulation and regranulation (15). This synchrony provides a unique temporal window through which neuro-immune dynamics can be non-invasively monitored using pruritus as a alternative functional indicator.

### 2.2 Neural pathways underlying pruritus in urticaria

**Figure 1:**
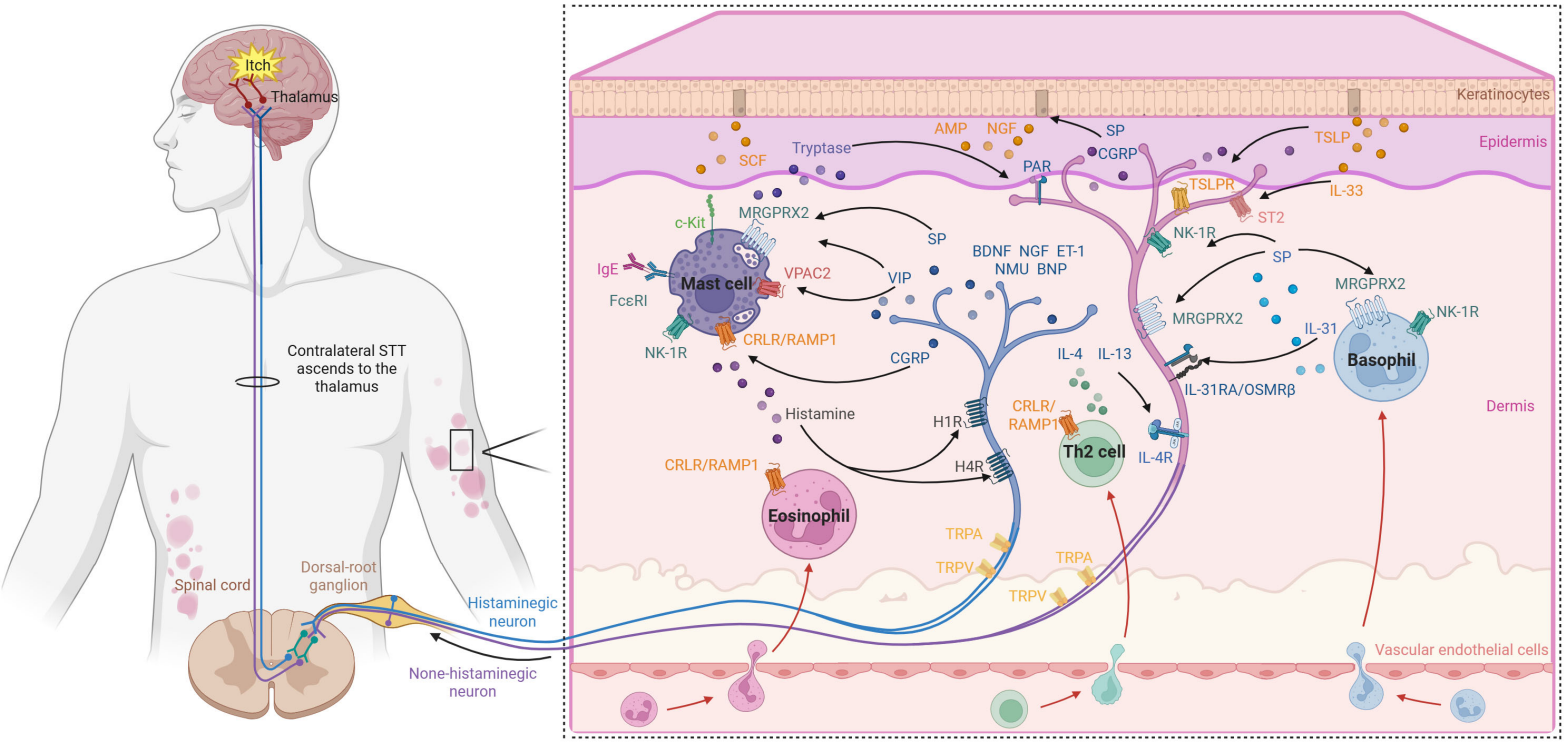
Neuroimmune interactions in urticaria. In urticarial skin lesions, immune cells such as mast cells, granulocytes, and Th2 cells are aberrantly activated, releasing a variety of bioactive substances including histamine, cytokines, and proteases. These mediators can directly excite or sensitize cutaneous sensory nerve endings, leading to the generation of action potentials. The electrical signals are transmitted via afferent nerves to the dorsal root ganglion (DRG) and the spinal cord, ultimately ascending through the spinothalamic tract to the cerebral cortex, where the sensation of itch is perceived. Concurrently, the activated sensory nerve endings release neuropeptides (e.g., SP, CGRP), which in turn promote further immune cell recruitment and degranulation. AMP, Antimicrobial Peptides; BDNF, Brain-Derived Neurotrophic Factor; BNP, Brain Natriuretic Peptide; CGRP,Calcitonin Gene-Related Peptide; cKit, Mast/stem cell growth factor receptor Kit; CRLP/RAMP1, Calcitonin Receptor-Like Protein/Receptor Activity-Modifying Protein 1; ET-1, Endothelin-1; FcϵRI, Fc Epsilon Receptor I; H1R, Histamine Receptor 1; H4R, Histamine Receptor 4; IL-4, Interleukin-4; IL-4R, Interleukin-4 Receptor; IL-13, Interleukin-13; IL-31, Interleukin-31; IL-33, Interleukin-33; MRGPRX2, Mas-Related G-Protein-Coupled Receptor X2; NGF, Nerve Growth Factor; NK1-R, Neurokinin-1 Receptor; NMU, Neuromedin U; PAR, Protease-Activated Receptor; SCF, Stem Cell Factor; SP, Substance P; ST2, Interleukin-1 Receptor-Like 1; TRPA, Transient Receptor Potential Ankyrin; TRPV, Transient Receptor Potential Vanilloid; TSLP, Thymic Stromal Lymphopoietin; TSLPR, Thymic Stromal Lymphopoietin Receptor; VIP, Vasoactive Intestinal Peptide; VPAC2, Vasoactive Intestinal Peptide Receptor 2.

Pruriceptors are functionally categorized into histaminergic and non-histaminergic subsets according to their specific ligands (**Figure 1**). Histaminergic pruritus is triggered by histamine released from mast cells and basophils, which act on neuronal Interleukin-1 Receptor (H1R) and Interleukin-4 Receptor (H4R) receptors to initiate downstream signaling through Phospholipase C (PLC)/Phospholipase A2 (PLA) - Transient Receptor Potential Vanilloid 1 (TRPV1)/Transient Receptor Potential Ankyrin 1 (TRPA1) pathways. In contrast, non-histaminergic pruritogens – including Interleukin-31 (IL-31), tryptase, bradykinin, lysophosphatidic acid (LPA), nerve growth factor (NGF), prostaglandin E_2_ (PGE_2_), endothelin-1 (ET-1), and substance P (SP) – activate their respective receptors (IL-31RA/OSMRβ, Protease-Activated Receptor-2/4 (PAR-2/4), Mas-Related G-Protein-Coupled Receptor X2 (MRGPRX2, and its murine counterpart MRGPRB2), Tropomyosin receptor kinase A (TrkA), Prostaglandin E2 receptor (EP), Endothelin receptor type A/Endothelin receptor type B (ETA/ETB), and Neurokinin-1 receptor (NK1R)) (16–22). These pathways converge on the phosphorylation of TRPV1/TRPA1 channels via JAK (Janus kinase)–STAT (Signal transducer and activator of transcription), Protein kinase C (PKC), or ERK (Extracellular signal-regulated kinase) signaling, leading to neuronal depolarization and action-potential generation.

Of note, MRGPRX2 is expressed not only on Dorsal Root Ganglia (DRG) neurons, where it participates in itch perception, but also on immune cells such as mast cells and basophils (23, 24). Owing to its relatively shallow and open ligand-binding pocket, MRGPRX2 is capable of recognizing and binding a diverse array of pathological mediators, including neuropeptides (e.g., SP), drugs (e.g., fluoroquinolones), defensins, and endogenous metabolites (e.g., cortistatin) (24). This broad-spectrum sensing property enables MRGPRX2 to transduce complex neurogenic stimuli within the CSU microenvironment into intracellular biochemical cascades. This receptor is currently a central focus of research into non-IgE-mediated degranulation pathways. Upon activation, it triggers the release of tryptase, IL-31, and Tumor Necrosis Factor (TNF-α), thereby establishing a “neuropeptide–mast cell–sensory nerve” positive feedback loop that operates independently of the classical IgE–FcϵRI (High-affinity IgE receptor, Fc fragment of IgE receptor I) axis. Studies have shown that mast cells in the skin of CSU patients exhibit significantly upregulated MRGPRX2 expression, contributing to heightened mast cell sensitivity (25). In a subset of CSU patients, a gain-of-function mutation in the MRGPRX2 gene (185A>G) has been identified, which increased degranulation, augmented calcium influx, and elevated phosphorylation of downstream ERK signaling molecules—ultimately rendering mast cells more susceptible to activation (26). Patients carrying this mutation also present with significantly higher disease activity scores (UAS7) and more severe clinical manifestations. Nevertheless, the precise role of MRGPRX2/B2 in itch pathogenesis in urticaria, its specific endogenous ligand profile, and its relative contribution to the complex itch network remain to be elucidated in future studies.

Pruriceptive signals are transmitted by polymodal, unmyelinated C-fibers and thinly myelinated Aδ-fibers, whose free endings reside at the dermo-epidermal junction (27). Their cell bodies are located in the ipsilateral DRG, where central projections synapse with second-order neurons in the spinal dorsal horn. These neurons then ascend via the spinothalamic tract to cortical regions such as the anterior cingulate cortex and insulars, where the sensory perception of itch is ultimately processed (18, 23, 27).

### 2.3 Neurosensitization in urticaria

Chronic pruritus is associated with a maladaptive decrease in itch threshold and an exaggerated sensory response - termed pruriceptive sensitization (28), representing a neuroplastic state in which previously subthreshold stimuli become more prominent. This process involves the upregulation of TRPV1/TRPA1 plasma channels or histamine expression in peripheral sensory fibers (29), the attenuation of the function of Neuropeptide Y (NPY)+ inhibitory neurons in the spinal cord, and hyperactivation of the emotional-related cortical circuit (30, 31). Collectively, these alterations can lead to the induction or exacerbation of itch perception even in response to minor stimuli.

In urticaria, particularly CSU, wheals and intense itching can be provoked by cold exposure, emotional stress, or mechanical friction, features that parallel the peripheral and central sensitization seen in chronic pruritus disorders such as atopic dermatitis. Functional magnetic resonance imaging (fMRI) studies in CSU patients have demonstrated enhanced functional connectivity between the left inferior orbitofrontal gyrus (ORBinf-L) and the caudate nucleus (32), correlating positively with itch severity. Conversely, reduced connectivity with the hippocampus has been inversely associated with anxiety levels, indicating a distinct reorganization of brain networks in chronic urticarial pruritus. Mast cells, Group 2 Innate Lymphoid Cells (ILC2s), and T helper type 2 cells (Th2 cells) release various cytokines such as IL-4, IL-13, thymic stromal lymphopoietin (TSLP), osteopontin, IL-31, IL-25, and IL-33, which directly activate primary sensory neurons and modulate neuronal responsiveness to other pruritogens (33). Notably, mast cell-derived IL-33 has been found to markedly enhance histamine-induced itch through IL-13-dependent mechanisms (34), establishing a detrimental cycle of “immune-nerve signal amplification” that represents a crucial peripheral driver of nerve sensitization and sustained pruritus in urticaria.

## 3 Neuroimmune features of urticaria subtypes

The onset of acute urticaria is primarily driven by the classical IgE/FcϵRI pathway activated by exogenous allergens. Although this process is often accompanied by alterations in the levels of various neuropeptides such as Vasoactive Intestinal Peptide (VIP), β-endorphin, somatostatin, and bombesin, neurons at this stage primarily act as recipients of downstream signals (35). An immunoelectron microscopy study of drug-induced acute urticaria revealed that both tryptase and coagulation factor XIII (FXIIIa) could be detected within superficial cutaneous nerves (36). This finding suggests that neural structures may be directly exposed to the protease-rich microenvironment resulting from mast cell degranulation, serving as local targets for active molecules such as tryptase, and thereby participating in signal reception and transmission during the acute phase.

Neuroimmune interactions could be considered key drivers of urticaria chronification. As understanding of the heterogeneity in mast cell activation mechanisms deepens, research focus is shifting from the clinical heterogeneity and variable treatment responses observed in CSU toward an endotype classification grounded in molecular mechanisms. This classification primarily includes IgE-mediated type I (autoallergy) and IgG-mediated type IIb (autoimmune) endotypes (37, 38). Studies have shown that IgE-mediated mast cell activation exhibits a “delayed but sustained” compound exocytosis pattern, forming large, irregular granule clusters, with histamine serving as the primary pruritogenic mediator (39). Accordingly, these type I CSU patients, who often present with elevated total IgE levels, show rapid responses to antihistamines and omalizumab (40).

In contrast, patients with type IIb CSU, as well as those who fit neither the type I nor type IIb endotype, demonstrate limited therapeutic responses to antihistamines and omalizumab (37, 41). This phenomenon is likely attributed to the involvement of non-IgE pathways. Unlike IgE-mediated activation, which primarily involves the release of pre-formed inflammatory mediators such as histamine and tryptase, non-IgE-mediated activation predominantly leads to the secretion of large quantities of newly synthesized cytokines and chemokines, resulting in non-histaminergic itch (37, 42). This may represent a core mechanism underlying the poor response to antihistamines and the chronicity observed in non-type I patients. Further metabolic studies indicate that non-IgE stimuli (e.g., neuropeptides or nanoparticles) can induce mitochondrial dysfunction, rapidly depleting glycolytic reserve and leading to a state of “metabolic stress” characterized by loss of metabolic flexibility (43). This process resembles a stress response to a neuroimmune alarm. SP released by sensory neurons can efficiently activate mast cells through this mechanism, and mediators released by activated mast cells can, in turn, further sensitize nerves, thereby establishing a self-sustaining, IgE-independent feedback loop.

Compared to CSU, the symptoms of Chronic Inducible Urticaria (CIndU) are triggered by specific, reproducible physical or environmental stimuli, exhibiting a more defined “stimulus-response” pattern (44, 45). Although the exact initiating mechanisms have yet to be fully elucidated, existing evidence—including passive transfer tests and the efficacy of barzolvolimab—strongly implicates mast cell activation and histamine release as central to its pathogenesis (46, 47). In CIndU, neuroimmune interactions might play a more pivotal “triggering” role, mirroring an acute response model of “stimulus–sensory nerve–immune effector.” Specific physical stimuli (e.g., cold, friction) can directly activate corresponding sensory nerve receptors, rapidly releasing neuropeptides via local axon reflexes (41, 48, 49). These neuropeptides can subsequently activate pathways such as MRGPRX2, initiating a rapid mast cell response.

## 4 Critical neuromediators and neuroactive peptides in urticaria

The pathogenesis of urticaria is driven by a complex interplay rather than a single pathogenic factor. Neuroactive mediators interact bidirectionally with immune cells, forming intricate neuroimmune communication networks. However, large-scale prospective studies validating the association between specific molecular pathways and disease severity remain conspicuously absent in urticaria, and no relevant targets with well-defined mechanisms have yet advanced to the stage of clinical translation. Nonetheless, the investigation of these molecules holds considerable value, whether as potential future therapeutic targets or as biomarkers for disease prognosis. Accordingly, based on the robustness of their clinical association with urticaria and the level of current research interest, we summarize, in rank order, the neuromediators and neuropeptides that have emerged as pivotal contributors to the pathophysiology of urticaria (**Table 1**).

**Table 1:** Important neuromediators and neuropeptides in urticaria.

Neuropeptide	Expression level	Action mechanism	Direct evidence in urticaria
SP	Increased (50–53)	Induces vasodilation and increases vascular permeability (54)	Elevated SP correlates with disease activity (51)
	No difference (55)	Chemoattracts basophils and mast cells (56, 57)	Intradermal injection elicits stronger W/F response (CIU, DPU) (58)
	Decreased (59)	Triggers mast-cell and basophil degranulation (39, 50)	
CGRP	Serum CGRP: pre- H1-antihistamine/omalizumab < post-treatment (60, 61)	Provokes vasodilation and potentiates SP-mediated plasma extravasation (59)	Intradermal injection induces W/F response (CIU, DPU) (58)
	CGRP^+^ cells in tissue: lesional > non-lesional (62)	Recruits CD4^+^, CD8^+^ T lymphocytes, eosinophils	↑ CGRP^+^ inflammatory infiltrate in lesional skin, composed mainly of elastase^+^ neutrophils, CD31^+^ cells, and MBP^+^ eosinophils (62)
		Drives Th2-polarized T-cell responses (63)	
		Modulates mast-cell activation (64, 65)	
BDNF	Serum & skin: ↑ (66)	Chemoattracts eosinophils	–
NGF	Serum: ↑ (67)	Associated with therapeutic resistance	Baseline ↓ in loratadine non-responders; ↑ after effective treatment (68)
		Regulates mast-cell functional behavior	
ET-1	Blood: ↑ (69)	Induces pruritus Promotes mast-cell degranulation	High ET-1 level characterizes antihistamine-resistant (AHR) CSU (69)
VIP	Serum: ↑ (trend, non-significant) (70, 71)	Promotes mast-cell degranulation	Intradermal injection potentiates wheal response; serum level shows upward trend (70, 71)
NMU	–	Modulates itch sensation	–
		Orchestrates Th2-type immunity	
BNP	–	Evokes pruritus	–

BDNF, Brain-Derived Neurotrophic Factor; BNP, Brain Natriuretic Peptide; CGRP,Calcitonin Gene-Related Peptide; CSU, Chronic Spontaneous Urticaria; CIU, Chronic Idiopathic Urticaria; DPU, Delayed Pressure Urticaria; ET-1, Endothelin-1; NGF, Nerve Growth Factor; NMU, Neuromedin U; SP, Substance P; VIP, Vasoactive Intestinal Peptide.

### 4.1 Substance P

SP is predominantly related from sensory nerve terminals following stimulation by histamine and leukotrienes, although it can also be produced by various immune cells (54). While the circulating SP levels in patients with CSU persists remain a matter of debate (**Table 1**), a consistent finding is the heightened cutaneous responsiveness to SP: intradermal SP administration induces more pronounced and persistent wheals, flares, itching, and granulocyte infiltration in individuals with CSU (54, 58).

SP exerts its biological effects primarily through two receptor classes: (1) the high-affinity NK1R, which is widely expressed on sensory neurons and immune cells including dendritic cells, eosinophils, basophils, and mast cells (72). In CSU, both SP and NK1R expression and markedly upregulated on basophils. SP not only recruits basophils via NK1R but also stimulates them to release histamine at levels comparable to those induced by anti-IgE and N-formylmethionyl-leucyl-phenylalanine (fMLP) (50, 56). (2) MRGPRX2: Fujisawa et al. identified MRGPRX2 as the receptor mediating SP-induced histamine and prostaglandin D2 (PGD2) release in cultured skin-derived mast cells (73). Upon neuronal release, SP activates MRGPRX2 on mast cells, leading to degranulation and subsequent release of inflammatory mediators, thereby establishing a reciprocal neuropeptide-mast cells feedback loop (48). MRGPRX2 expression is elevated in severe CSU patients and correlates with disease severity (74). Prolonged SP exposure further elevate MRGPRX2 expression amplifying this loop (57).

SP exerts concentration-dependent effects on mast cells, eliciting chemotaxis at low concentrations and degranulation at high concentrations (57). In murine models, SP reproduces urticaria-like symptoms, which can be mitigated by inhibiting mast cell degranulation through the Lyn/PLC-p38 signaling pathway (artemisinic acid) or by Src kinase phosphorylation (paeonol) (75, 76). However, the precise contribution of MRGPRX2 signaling to human urticaria pathogenesis requires further investigation.

### 4.2 Calcitonin gene-related peptide

CGRP is secreted by nociceptive C fibers and other sensory neurons via calcium-dependent exocytosis following TRPV1 activation (77). CGRP acts via a heterodimeric receptor complex composed of calcitonin receptor-like receptor (CRLR) and receptor activity-modifying protein 1(RAMP1), and influences various immune and structural cells, including T cells, B cells, macrophages, mast cells, and dendritic cells (**Table 1**) (65).

CGRP is a potent vasodilatory properties, as evidenced by injection studies showing enhanced wheal (W) and erythema (F) responses in patients with chronic idiopathic urticaria (CIU) and delayed pressure urticaria (DPU) (58). Additionally, CGRP functions as a neuropeptide that induces chemotaxis of CD4^+^ and CD8^+^ T lymphocytes and promoting eosinophil migration (78, 78). Mast cells have been observed in close proximity to CGRP+ nerve fibers (79, 80). CGRP exhibits dual effects on mast cells, it can directly trigger histamine release via degranulation (64), while paradoxically suppressing IgE-dependent degranulation in primary human mast cells *in vitro* (65). Moreover, CGRP receptors activation the Cyclic adenosine monophosphate/Protein kinase A (cAMP/PKA) pathway, driving Th2 polarization of T cells through Cluster of Differentiation 3/28 (CD3/CD28) costimulation (63).

### 4.3 Neurotrophins

Neurotrophins exert their biological effects through two receptor classes: the high-affinity Trk isoforms and the low-affinity pan-neurotrophin receptor p75 (p75NTR). Both receptor sets are expressed not only on sensory neurons but also on keratinocytes, melanocytes, various myelocytes, immature and mature human mast cells, among others (81, 82). The paragraphs below focus on the family members most frequently implicated in urticaria (summaried in **Table 1**).

#### 4.3.1 Brain-derived neurotrophic factor

Elevated serum and cutaneous BDNF levels have been observed in patients with CSU compared to healthy or non-allergic individuals (66). Eosinophils are a significant source of BDNF, promotes their chemotaxis and inhibits apoptosis, contributing to their accumulation in lesional skin (72, 83, 84). Keratinocytes also produce and retain BDNF, likely upregulated by TNF-α, thereby augmenting local BDNF concentrations in affected skin (85–87).

#### 4.3.2 Nerve growth factor

Bonini et al. reported elevated serum levels of NGF in patients with urticaria (67). Conversely, its low-affinity receptor p75NTR is down-regulated on perivascular cells in both affected and unaffected skin of patients with delayed pressure, acute, or chronic urticaria (88). As NGF shares several functions with stem cell factor (SCF) - including mast cell proliferation, differentiation, survival, chemotaxis and mediator release, this downregulation may represent a negative feedback to increased mast cell activity. Additionally, Ozseker et al. observed that CSU patients unresponsive to loratadine exhibit significantly lower baseline NGF levels than responders or healthy controls (68). Symptom improvement correlates with increased NGF levels, indicating a possible association between lower serum NGF levels and treatment resistance in CSU patients.

### 4.4 Endothelin-1

ET-1 evokes pruritus through direct activation of neuronal ETA receptors and simultaneously amplifies IgE-dependent mast-cell degranulation (89, 90). Murine models demonstrate that ET-1 exacerbates anaphylactic shock and urticaria-like reactions. Clinically, serum ET-1 levels are significantly elevated in patients with CSU, particularly in those resistant to antihistamines therapy (69), positioning ET-1 as a potential predictor of therapeutic refractoriness.

### 4.5 Vasoactive intestinal peptide

VIP released by DRG neurons, induces mast cell degranulation through Vasoactive intestinal peptide/pituitary adenylate cyclase-activating polypeptide receptor 2 (VPAC2) and/or MRGPRX2 receptors on mast cells (91, 92). CSU patients exhibit increased wheal responses to intradermal VIP injections and a non-significant trend towards elevated serum VIP levels (70, 71). Altered VIP levels in patients with chronic urticaria complicated by allergic rhinitis may hold clinical importance. Collectively, the trio of VIP, SP, and neuropeptide Y has been proposed as biomarkers panel for assessing disease severity and treatment efficacy in CSU (17).

### 4.6 Other potential neuropeptides

Several additional neuropeptides have been explored in the context of allergic and pruritic disorders. Neuromedin U (NMU) demonstrated dual functionality: excitatory neurons expression NMUR2 receptors in the dorsal horn are critical for mechanical itch, transmission, whereas NMU also enhance Th2 immune responses by regulating ILC2 (93–95).

Another classical example brain natriuretic peptide (BNP), a pruritogenic mediator that acts through both gastrin-releasing peptide (GRP)-dependent and independent mechanism (96, 97). Increased levels of BNP and its receptors have been detected in chronic pruritic conditions such as atopic dermatitis (98). However, the precise contributions of these neuropeptides to urticaria remain to be fully elucidated.

## 5 Neuro-immune cellular axis in urticaria

**Figure 2:**
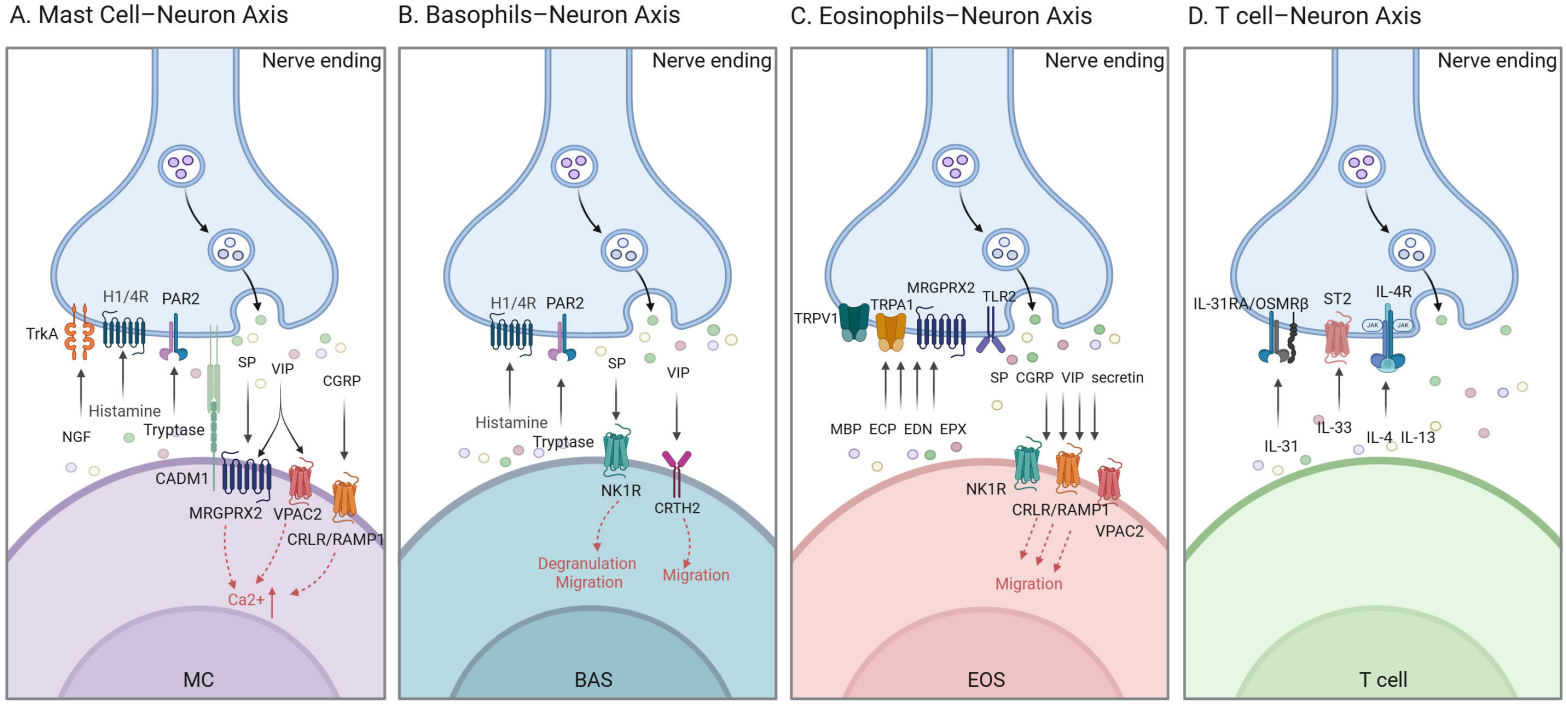
Neuro-immune cellular axis in urticaria. **(A)** CADM1 mediates the adhesion of MCs to sensory nerve fibres. MCs release histamine, tryptase and NGF, which activate H1/H4R, PAR2 and TrkA on nerve endings, respectively, thereby initiating pruritic signaling. In turn, neuropeptides SP, VIP and CGRP engage MRGPRX2, VPAC2 and CRLR/RAMP1 on MCs, evoking Ca²^+^ influx that positively feeds back to amplify degranulation. **(B)** Bas exhibit an expression profile analogous to that of MCs. In addition, SP (via NK1R) and VIP (via CRTH2) released from nerve terminals act synergistically to promote Bas chemotaxis toward the skin, exacerbating local inflammation. **(C)** Eos-derived granule proteins (ECP, MBP, EPX and EDN) activate sensory neurons through MRGPRX2, TLR and TRPV1/TRPA1 channels, transducing pruriceptive signals. Conversely, neuropeptides SP (NK1R), CGRP (CRLR/RAMP1), VIP (VPAC2) and secretin released by nerve endings enhance Eos tissue infiltration, establishing a self-amplifying feedback loop. **(D)** T cell-derived cytokines IL-31, IL-4, IL-13 and IL-33 engage neuronal IL-31R/OSMRβ, IL-4R and ST2 receptors to induce pruritus transmission and trigger neurogenic inflammation. Bas, Basophils; CADM1, Cell Adhesion Molecule 1; CGRP, Calcitonin Gene-Related Peptide; CRLP/RAMP1, Calcitonin Receptor-Like Protein/Receptor Activity-Modifying Protein 1; CRTH2, Chemoattractant Receptor-Homologous Molecule Expressed On Th2 Cells; ECP, Eosinophil Cationic Protein; EDN, Eosinophil-Derived Neurotoxin; Eos, Eosinophils; EPX, Eosinophil Peroxidase; H1/H4R, Histamine Receptor 1/4; IL-31R/OSMRβ, Interleukin-31 Receptor/Oncostatin M Receptor β; IL-4R, Interleukin-4 Receptor; MC, Mast Cell; MBP, Major Basic Protein; MRGPRX2, Mas-Related G-Protein-Coupled Receptor X2; NGF, Nerve Growth Factor; NK1R, Neurokinin-1 Receptor; PAR2, Protease-Activated Receptor 2; SP, Substance P; ST2, Interleukin-1 Receptor-Like 1; TLR, Toll-Like Receptor; TrkA2, Tropomyosin Receptor Kinase A2; TRPA1, Transient Receptor Potential Ankyrin 1; TRPV1, Transient Receptor Potential Vanilloid 1; VIP, Vasoactive Intestinal Peptide; VPAC2 Vasoactive Intestinal Peptide Receptor 2.

Although neuroimmune crosstalk in urticaria involves a complex network of multiple components and pathways, this review adopts an axis based framework to better elucidate the bidirectional interactions between immune cells and neurons and to highlight the distinct contributions of different immune cell types (**Figure 2**).

### 5.1 Mast cell–neuron axis

Mast cell numbers were elevated in all urticaria subtypes, affecting both in lesional and non-lesional skin (99). Sensory nerve endings within the skin—particularly unmyelinated C fibers—are densely distributed in the papillary dermis and often intercalate between keratinocytes (100–102). Keratinocytes contribute to this architecture by secreting SCF, especially its soluble isoforms, thereby establishing a chemotactic microenvironment in the papillary dermis that guides the homing of mast cell precursors (103, 104). Recruited mast cells subsequently reside in close proximity to nerves, blood vessels, and the epidermis, forming physical appositions with sensory nerve endings (105). Beyond its role in chemotaxis, the binding of SCF to the c-Kit receptor on mast cells activates Phosphoinositide 3-kinase (PI3K) and Mitogen-activated protein kinase (MAPK) signaling pathways (106, 107). This not only promotes mast cell survival within urticarial lesions but also lowers their activation threshold, thereby enhancing IgE-mediated degranulation (108). Additionally, the adhesion molecule Cell Adhesion Molecule 1 (CADM1) enhances mast cells-nerve fibers interactions (109–112), and it appears to enhance mast cell sensitivity to neural stimuli, suggesting that structural proximity translates into functional sensitivity (113–115).

Mast cells are categorized into two subsets according to their neutral protease expression profiles: MC_T_ (expressing only tryptase) and MC_TC_ (co-expressing tryptase and chymase) (116–118). Notably, the MC_TC_ subset exhibits a non-IgE-dependent activation mechanism, allowing direct degranulation in response to stimuli such as neuropeptides and certain drugs. In the dermis of patients with CSU, MC_TC_ constitutes over 99% of the mast cell population, positioning it as a key effector cell under neurogenic regulation (118). Mast cell phenotypes are dynamically modulated by microenvironmental cues, and this plasticity significantly influences their repertoire of neurotransmitter receptor expression (119). Studies have demonstrated spatial heterogeneity in receptor distribution among mast cells within the same tissue. For instance, MRGPRX2 is expressed exclusively by proximal lung mast cells, whereas distal lung mast cells lack its expression, thereby determining the specificity of their response to neurogenic activation signals (120). Furthermore, neural factors, as integral components of the microenvironment, regulate functional characteristics of mast cells, including Toll-like receptor expression levels and sensitivity to neuropeptides (119, 121). In the pathological context of CSU, the cutaneous microenvironment fosters a mast cell population predominantly skewed toward the MC_TC_ subset. This subset not only expresses neuropeptide receptors such as MRGPRX2 but also retains the capacity for non-IgE-dependent degranulation, rendering it a central player in neuro-immune crosstalk. Elucidating the mechanisms by which the neuronal microenvironment shapes mast cell phenotypes in urticaria may offer critical insights into disease pathogenesis and pave the way for precision therapeutic strategies.

Mast cell-derived mediators exert multifaceted effects on sensory neurons, playing a pivotal role in the neuroimmune regulation of itch. Histamine primarily induces peripheral itching through H1R and H4R, triggering phospholipase A2 (PLA2) and 12-lipoxygenase signaling cascades, subsequently activating TRPV1+ sensory neurons, predominantly C fibers, to elicit pruritus. In parallel, trypsin activates protease-activated receptor 2 (PAR2) on neurons, initiating phospholipase Cβ (PLCβ) and PKC pathways, which open TRPV1 channels and promote the release of SP and CGRP (122). NGF binding to the TrkA receptor on the neuronal surface reduces excitation threshold, leading to the synthesis and release of neuropeptides such as substance P, CGRP, and BDNF (123). In specific microenvironments or upon activation, mast cell-derived cytokines (IL-4, IL-13, IL-31, TNF-α) ligate cognate receptors (IL-4Rα/IL-13Rα1, IL-31RA/OSMR, Tumor necrosis factor receptor1/2 (TNFR1/2)) on sensory neurons, thereby sustaining neurogenic inflammation and chronic itch (124–126).

Conversely, mast cells express various neuropeptide receptors (such as NK-1, MRGPRX2, VPAC-1/2, and CGRP receptors) and are regulated by neurons. Co-culture studies have shown that SP released from activated nerve endings influences mast cells in a dose-dependent impact. High concentrations directly induce mast cell degranulation, while low concentrations sensitize the cells, reducing the degranulation threshold for subsequent stimulation (127). Initially, this effect was attributed to NK-1R mediated signaling. Bone marrow-derived mast cells (BMMCs) cultured with IL-4 and stem cell factors showing increased sensitivity to SP through the expression of functional NK-1 receptors (128). However, recent research had identified MRGPRX2/B2 as the primary receptor for substance P-induced mast cell activation (129, 130). MRGPRB2-mediated degranulation predominantly releases histamine and broadly activates specific nonpeptide itch receptor neurons (Mrgprd+, Mrgpra3+, 5ht1f+), shedding light on the limited efficacy of histamine antagonists in certain chronic itch conditions (23). Studies on MRGPRB2-deficient mice have demonstrated a significant reduction in pruritus in allergic inflammatory models (131). The clinical inefficacy of NK-1R antagonists in conditions such as asthma, migraine, and chronic pruritus further underscores the critical role of the MRGPRX2/B2 pathway in the SP-mast cell axis (132). These findings provide a mechanistic rationale for the limited clinical efficacy of H1-antihistamines and NK-1R antagonists in certain chronic itch conditions, highlighting the MRGPRX2/B2 axis as a key driver of SP–mast cell signaling.

Except SP, mast cells exhibited significant heterogeneity responses to other neuropeptides as well. For instance, CGRP does not induce degranulation in the human mast cell line LAD2, despite activating functional CGRP1 receptors in murine BMMCs (91). While CGRP evokes intracellular Ca²^+^ mobilization and selective release of mast cell protease-1 (mMCP-1), it fails to elicit full degranulation (133). The precise functional profile of CGRP on mast cells derived from urticaria patients remains to be defined. The interplay between VIP and mast cells is intricate. VIP can trigger mast cell degranulation *in vitro* through VPAC2 or MRGPRX2 receptors, yet *in vivo* studies suggest a potential stabilizing effect (134, 135). Moreover, neuronal co-culture modulates mast cell phenotypes, increasing granule content, FcϵRI expression, and PGD2 receptor Chemoattractant Receptor-Homologous Molecule Expressed On Th2 Cells (CRTH2) expression (134, 136).

Importantly, mast cell–neuron interactions should not be viewed in isolation. Other immune cells, such as basophils, actively contribute to the amplification of neuroimmune signaling. Basophils upregulate tetrahydrobiopterin (BH4) enhances mast cell secretion of histamine and serotonin and directly activates neuronal TRPA1 channels, intensifying pruritus (137). Collectively, these findings underscore a dynamic bidirectional communication network among mast cells, sensory neurons, and immune effector cells that underlies neurogenic inflammation and chronic itch in urticaria.

### 5.2 Granulocyte–neuron axis

Under physiological conditions, granulocytes are rarely found in close proximity to sensory neurons. Nevertheless, in the pathological condition of CSU, particularly in individuals exhibiting basopenia, granulocytes may migrate to the skin in response to chemokines enabling their spatial and functional interaction with peripheral nerve endings. This phenomenon of granulocyte co-localization with sensory nerves has been documented in the afflicted skin of patients with allergic and chronic pruritic disorders, implicating granulocytes as active participants in neuroimmune communication (138).

Activated eosinophils release a variety of cationic and cytotoxic granular proteins, including major basic proteins (MBP), eosinophil cationic protein (ECP), eosinophil peroxidase, and eosinophil derived neurotoxin (EDN). These mediators can directly engage neuronal receptor such as MRGPRX2 and Toll-like receptor 2 (TLR2), enhancing sensory neurosensitivity, promoting nerve growth, and increasing neuropeptide synthesis (139). This mechanistic interplay may account for the positive correlation between serum eosinophil granule protein levels and urticaria severity (140–142). Conversely, neuropeptides like SP, CGRP, secretoneurin, VIP, and secretin released by sensory nerves can stimulate eosinophil migration (78). This bidirectional relationship is further supported by findings that eosinophil depletion reduces SP levels around cutaneous nerves and diminishes scratching behavior in a mouse model of repeated sensitization, indicating that eosinophils contribute to itching by promoting cutaneous innervation (143).

Basophils exhibit function similarities to mast cells, involving degranulation to release inflammatory mediators and pruritus-inducing factors such as histamine, neuropeptide P, NGF, Th2 cytokines (e.g., IL-4, IL-13, IL-31, and TSLP), PGD2, PGE2, and proteases (e.g., cathepsin S) (144). Upon allergen stimulation, basophils migrate to the skin and interact with sensory nerve fibers, as evidenced in murine models (145). Notably, basophils derived from CSU patients exhibit elevated levels of SP and its receptor NK1R (50). SP not only facilitates basophil recruitment in the bloodstream through NK1R binding but also triggers degranulation and histamine release, thereby amplifying neurogenic inflammation. Cima et al. demonstrated that NK1R antagonists effectively impede basophil migration, suggesting that SP orchestrates their directed chemotaxis via NK1R signaling (56, 134). Furthermore, basophils express CRTH2, which may also regulate their chemotaxis.

CSU patients can be categorized into three functional subsets based on basophil responsiveness to IgE stimulation: responders, non-responders, and basopenics. Among these, basopenic patients exhibited the highest pruritus intensity scores (146). Despite this, systematic comparisons of neuroimmune communication patterns across these subgroups remain limited. Given that the upregulation of the MRGPRX2 receptor on basophils is induced by IgE/FcϵRI signaling and its activation is directly linked to pruritus (147). Further exploration of MRGPRX2-mediated basophil–neuron interactions could provide valuable insight into CSU pathogenesis and identify potential therapeutic targets, especially for patients with antihistamine-refractory disease.

### 5.3 T-cell–neuron axis

The T cell-neuron axis is notable for its secretion of Th2 cytokines, particularly IL-31, which can directly induce pruritus by binding to the IL-31RA/OSMRβ dimer on sensory neurons and activating STAT3 signaling (148). Intradermal administration of IL-31 elicits immediate itch responses, underscoring its capacity to stimulate target neurons (149). In CSU, elevated serum IL-31 levels correlate positively with itch severity, disease activity, and impaired quality of life (150). Higher IL-31 levels are observed in antihistamine nonresponders and decrease following successful omalizumab treatment (151). Beyond its sensory effects, IL-31 also modulates inflammation by altering microRNA expression profiles, including miR-221, and by promoting IL-4 and IL-13 production from IgE- or IL-3-stimulated eosinophils, establishing a Th2-dominant inflammatory feedback loop (150, 152).

Both IL-4 and IL-13 can directly activates sensory neurons, with IL-4R α and IL-13R α1 receptors identified in mouse and human DRG. Experimental IL-4 administration induces scratching behavior through the IL-4Rα/JAK1 signaling pathway, highlighting a mechanistic link between Th2 cytokines and neuronal hypersensitivity in urticaria (125).

In addition to adaptive T cells, Innate lymphoid cells (ILCs) have recently been identified as neuro-immune intermediaries. Cholinergic neurons release the neuropeptide NMU, which activates ILC2 to enhance type 2 cytokine responses (153). Additionally, CGRP modulates ILC2 activation, influencing type 2 inflammation (154). While research has primarily focused on inflammatory bowel disease and allergic rhinitis, insights from studying urticaria are valuable. ILC2 cells reside in the skin as part of mucosal immunity and alterations in the local microenvironment can dynamically reshape their phenotype and function (155). For instance, in conditions like psoriasis triggered by IL-23 or imiquimod, the quiescent ILC2 population in the skin can transition into an ILC3-like subset, contributing to the immune response in psoriasis and exacerbating skin inflammation (156).

### 5.4 Additional cellular contributors

Neuropeptides (e.g.,SP and CGRP) have been shown to upregulate the expression of antimicrobial peptides (AMP) and NGF in keratinocytes, thereby contributing to the amplification of neurogenic inflammation in the skin (157–159). Conversely, TSLP, primarily secreted by keratinocytes, can independently activate TRPV1-sensory neurons to initiate itch signaling (160). In the dermis of CSU patient, there is a notable increase in TSLP+ cells and IL-33+ cells, encompassing endothelial cells, fibroblasts, macrophages, and mast cells (161). Elevated levels of serum TSLP and IL-33 accompanied by IL-33 gene single nucleotide polymorphisms (SNPs) linked to susceptibility to CSU (162, 163). Administration of TSLP induces robust scratching behavior in wild-type mice consistent with the expression of functional TSLP receptors in both mouse and human DRGs. In allergic contact dermatitis models, IL-33 directly activates sensory neurons through ST2 receptors, exacerbating itch during skin inflammation (164). These findings collectively suggest that epithelial-derived alarmins, such as TSLP and IL-33, serve as key neuroimmune mediators bridging barrier dysfunction and neuronal hypersensitivity. However, the precise mechanisms underlying TSLP/IL-33-driven neuronal activation in CSU remain to be elucidated. The molecular mechanisms underlying the “itch-scratch” cycle in atopic dermatitis, particularly those involving keratinocyte-derived mediators, are relatively well established (165). However, if, how, and to what extent these mechanisms contribute to urticaria and interact with mast cell-driven wheal responses remain to be elucidated.

## 6 Current therapeutic targets for urticaria

Neuroimmune interactions represent a promising and rapidly evolving therapeutic frontier, however, progress in urticaria remains limited. To date, aside from agents targeting IL-4/IL-13, the JAK-STAT signaling pathway, and MRGPRX2 antagonists—which have shown more established potential for clinical translation—most neuroactive molecules (e.g., SP, BDNF), although correlated with disease activity, have not yet advanced to the stage of clinical validation through targeted therapeutics, and their causal involvement awaits confirmation in further functional studies. In this section, we summarize emerging pharmacologic agents that modulate neuroimmune signaling in urticaria, with a particular focus on compounds currently under clinical evaluation for this indication (**Table 2**).

**Table 2:** Investigational agents that modulate neuroimmune signaling in urticaria.

Target	Mechanism	Drug	Urticaria type	Phase	Status	ClinicalTrials.gov identifier
TSLP	Anti TSLP	Tezepelumab	CSU	Phase 2	Completed	NCT04833855
IL-4Rα	Anti-IL-4Rα	Dupilumab	CSU	Phase 3	Completed	NCT05526521
			CSU	Phase 2	Completed	NCT03749135
			CSU	Phase 3	Completed	NCT04180488
			CholU	Phase 2	Completed	NCT03749148
			ColdU	Phase 3	Completed	NCT04681729
TNF	TNF blockers	Etanercept	CSU	Phase 2–3	Withdrawn	NCT01030120
IL-31	Anti-IL-31 (via OSMRβ)	Vixarelimab	CSU	Phase 2	Completed	NCT03858634
JAK	JAK1 inhibitor	Povorcitinib	CSU	Phase 2	Active, not recruiting	NCT05936567
	JAK1 inhibitor	TLL018	CSU	Phase 1	Completed	NCT05373355
	JAK1/3 inhibitor	Tofacitinib	CSU	An Open-Label, Investigator-Initiated, Single-Centre Pilot Study		
	JAK1/TYK2 inhibitor	Abrocitinib	CSU	Case series		
MRGPRX2	MRGPRX2 receptor	EP262	CIU	Phase 1b	Completed	NCT06050928
	(antagonist)	EP262	CSU	Phase 2	Terminated	NCT06077773
		EVO756	CSU	Phase 2b	Recruiting	NCT06873516
		EVO756	CIU	Phase 2	Completed	NCT06603220

CholU, Cholinergic Urticaria; CSU, Chronic Spontaneous Urticaria; ColdU, Cold Urticaria; CIU, Chronic Idiopathic Urticaria; IL-4R, Interleukin-4 Receptor; IL-31, Interleukin-31; JAK, Janus Kinase; MRGPRX2, Mas-Related G-Protein-Coupled Receptor X2; TNF, Tumor Necrosis Factor; TSLP, Thymic Stromal Lymphopoietin.

At present, CSU, especially antihistamine resistant and/or omalizumab low responder, is a major target for drug management. Dupilumab (anti-IL-4R α) and JAK inhibitors are currently the most widely studied biotherapies. In randomized, placebo-controlled, double-blind phase 3 trials, dupilumab significantly ameliorated disease activity (ISS7, UAS7, HSS7) and lowered total serum IgE in omalizumab-naïve patients with H1-antihistamine-refractory CSU (166, 167). However, the magnitude of IgE reduction correlated only weakly with clinical improvement, implying that dupilumab’s therapeutic effect is mediated predominantly through IL-4/IL-13–dependent suppression of B-cell clonal expansion and IgE class switching, with an additional contribution from IgE-independent pathways. JAK inhibitors abrogate the JAK-STAT signaling cascade, thereby interrupting convergent cytokine networks and attenuating pruritus via direct modulation of nociceptive neuron receptors (168). An investigator-initiated, open-label, single-center pilot study and complementary case series have demonstrated that oral tofacitinib elicits rapid and profound clinical responses in patients resistant to maximally dosed antihistamines, whereas abrocitinib confers substantial benefit in omalizumab-non-responsive individuals (169, 170).

In a recently completed multicenter, double-blind, randomized, placebo-controlled phase II trial, Tezepelumab - an mAb inhibiting TSLP, failed to achieve the primary endpoint at week 16 in the overall CSU cohort (171). Nevertheless, in omalizumab-naïve participants, tezepelumab produced a statistically significant and clinically meaningful reduction in UAS7 versus placebo, with therapeutic benefit persisting for up to 32 weeks after treatment cessation; this durability was not observed with omalizumab. *Post-hoc* analyses indicated that the sustained response was most evident in patients characterized by low baseline total IgE and a longer disease duration.

MRGPRX2 antagonists are rapidly emerging as a next-generation therapeutic strategy for refractory urticarias. The selective MRGPRX2 antagonist EP262 has been shown in MRGPRX2-KI mice to abolish agonist-evoked cutaneous mast-cell degranulation, providing the mechanistic rationale for its current advancement into phase II trials for both CIndU and CSU (172). More recently, PSB-172656 has demonstrated comparable efficacy in human mast-cell systems and complementary disease models. Collectively, these data signal that MRGPRX2-targeted intervention is transitioning from proof-of-concept to clinical translation (173).

## 7 Outlook

Investigation into the interplay between cutaneous sensory neurons and urticaria remains in its infancy with fundamental pathways, causal relationships, and therapeutic targets are still largely undefined. Future research could prioritize three pivotal unanswered questions: (1) Which mediators critically define urticaria subtypes, disease phases, and severity strata? (2) Within a precision-medicine framework, which mediator(s) or neuro-immune signaling axis(es) should be targeted to maximize clinical benefit? (3) How do key neuromediators—such as SP and BDNF—modulate to comorbid anxiety and depression in urticaria patients, and can integrated therapies therapeutic strategies be designed that simultaneously interrupt neuro-immune circuits and ameliorate psychiatric comorbidities?

Advanced molecular and cellular profiling, combined with single-cell sequencing, to map the spatiotemporal dynamics of neuro-immune circuits and pruritus sensitization will provide a comprehensive mechanistic understanding of urticaria and furnish a rational basis for development of more precise and effective therapeutic interventions.

## References

[1] Maurer M Zuberbier T Metz M . The classification, pathogenesis, diagnostic workup, and management of urticaria: An update. Handb Exp Pharmacol. (2022) 268:117–33. doi: 10.1007/164_2021_506. PMID: 34247278

[2] Zuberbier T Abdul Latiff AH Abuzakouk M Aquilina S Asero R Baker D . The international EAACI/GA^2^LEN/EuroGuiDerm/APAAACI guideline for the definition, classification, diagnosis, and management of urticaria. Allergy. (2022) 77:734–66. doi: 10.1111/all.15090. PMID: 34536239

[3] Maurer M Rosén K Hsieh HJ Saini S Grattan C Gimenéz-Arnau A . Omalizumab for the treatment of chronic idiopathic or spontaneous urticaria. N Engl J Med. (2013) 368:924–35. doi: 10.1056/NEJMoa1215372. PMID: 23432142

[4] Kolkhir P Giménez-Arnau AM Kulthanan K Peter J Metz M Maurer M . Urticaria. Nat Rev Dis Primers. (2022) 8:61. doi: 10.1038/s41572-022-00389-z. PMID: 36109590

[5] Sánchez-Borges M Ansotegui IJ Baiardini I Bernstein J Canonica GW Ebisawa M . The challenges of chronic urticaria part 2: Pharmacological treatment, chronic inducible urticaria, urticaria in special situations. World Allergy Organ J. (2021) 14:100546. doi: 10.1016/j.waojou.2021.100546. PMID: 34141049 PMC8188551

[6] Bernstein JA Kavati A Tharp MD Ortiz B MacDonald K Denhaerynck K . Effectiveness of omalizumab in adolescent and adult patients with chronic idiopathic/spontaneous urticaria: a systematic review of “real-world” evidence. Expert Opin Biol Ther. (2018) 18:425–48. doi: 10.1080/14712598.2018.1438406. PMID: 29431518

[7] Guillén-Aguinaga S Jáuregui Presa I Aguinaga-Ontoso E Guillén-Grima F Ferrer M . Updosing nonsedating antihistamines in patients with chronic spontaneous urticaria: a systematic review and meta-analysis. Br J Dermatol. (2016) 175:1153–65. doi: 10.1111/bjd.14768. PMID: 27237730

[8] Slominski A Wortsman J . Neuroendocrinology of the skin. Endocr Rev. (2000) 21:457–87. doi: 10.1210/edrv.21.5.0410. PMID: 11041445

[9] Kim B Rothenberg ME Sun X Bachert C Artis D Zaheer R . Neuroimmune interplay during type 2 inflammation: Symptoms, mechanisms, and therapeutic targets in atopic diseases. J Allergy Clin Immunol. (2024) 153:879–93. doi: 10.1016/j.jaci.2023.08.017. PMID: 37634890 PMC11215634

[10] Han C Zhu X Sokol CL . Neuroimmune circuits in allergic diseases. Annu Rev Immunol. (2025) 43:367–94. doi: 10.1146/annurev-immunol-082423-032154. PMID: 39977604 PMC12954524

[11] Kalogeromitros D Psaltopoulou T Makris M Koti I Chliva C Stefanadi E . Can internet surveys help us understanding allergic disorders? - Results from a large survey in urticaria in Greece. J Eur Acad Dermatol Venereol. (2011) 25:532–7. doi: 10.1111/j.1468-3083.2010.03817.x. PMID: 20704630

[12] Maurer M Ortonne JP Zuberbier T . Chronic urticaria: An internet survey of health behaviours, symptom patterns and treatment needs in European adult patients. Br J Dermatol. (2009) 160:633–41. doi: 10.1111/j.1365-2133.2008.08920.x. PMID: 19014398

[13] Fetzer SJ . Pruritus and urticaria. J Perianesth Nurs. (1999) 14:294–299, 318. doi: 10.1016/s1089-9472(99)80037-9. PMID: 10827639

[14] Brenaut E Garlantezec R Talour K Misery L . Itch characteristics in five dermatoses: Non-atopic eczema, atopic dermatitis, urticaria, psoriasis and scabies. Acta Derm Venereol. (2013) 93:573–4. doi: 10.2340/00015555-1599. PMID: 23722181

[15] Saxena DR Saxena DIG Sharma DP . Urticaria with homoeopathic management. Int J Hom Sci. (2020) 4:83–5. doi: 10.33545/26164485.2020.v4.i4b.257

[16] Bernstein JS Sussman G Pite H Bernstein JA . Advancements in novel therapeutics for chronic spontaneous urticaria. J Allergy Clin Immunol Pract. (2025) 13:2272–85. doi: 10.1016/j.jaip.2025.05.035. PMID: 40447049

[17] Wang Y Zhao G . Clinical values of serum neuropeptide changes in patients with chronic urticaria complicated with allergic rhinitis. Ir J Med Sci. (2024) 193:1137–42. doi: 10.1007/s11845-023-03553-w. PMID: 37874502

[18] Misery L Pierre O Le Gall-Ianotto C Lebonvallet N Chernyshov PV Le Garrec R . Basic mechanisms of itch. J Allergy Clin Immunol. (2023) 152:11–23. doi: 10.1016/j.jaci.2023.05.004. PMID: 37201903

[19] Hofman ZLM van den Elzen MT Kuijpers J de Maat S Hack CE Knulst AC . Evidence for bradykinin release in chronic spontaneous urticaria. Clin Exp Allergy. (2020) 50:343–51. doi: 10.1111/cea.13558. PMID: 31899843

[20] Lopes GPR Ramos YAL Pereira NV Sotto MN Kawakami JT Pincelli MS . Evaluation of pruritus biomarkers expression in chronic spontaneous urticaria. Arch Dermatol Res. (2025) 317:731. doi: 10.1007/s00403-025-04170-6. PMID: 40261414

[21] Vander Does A Ju T Mohsin N Chopra D Yosipovitch G . How to get rid of itching. Pharmacol Ther. (2023) 243:108355. doi: 10.1016/j.pharmthera.2023.108355. PMID: 36739914

[22] Dobrican-Băruța CT Deleanu DM Muntean IA Pintea I Florea CM Filip GA . IL-31-pruritus interleukin: Serum values and clinical impact in chronic spontaneous urticaria-a Romanian retrospective study. J Clin Med. (2023) 12:5957. doi: 10.3390/jcm12185957. PMID: 37762898 PMC10532079

[23] Meixiong J Anderson M Limjunyawong N Sabbagh MF Hu E Mack MR . Activation of mast-cell-expressed Mas-related G-protein-coupled receptors drives non-histaminergic itch. Immunity. (2019) 50:1163–71.e5. doi: 10.1016/j.immuni.2019.03.013. PMID: 31027996 PMC6531358

[24] Yang F Guo L Li Y Wang G Wang J Zhang C . Structure, function and pharmacology of human itch receptor complexes. Nature. (2021) 600:164–9. doi: 10.1038/s41586-021-04077-y. PMID: 34789875

[25] Metz M . No allergy, but mast cells are involved: MRGPRX2 in chronic inflammatory skin diseases. J Eur Acad Dermatol Venereol. (2025) 39:451–2. doi: 10.1111/jdv.20541. PMID: 39996342 PMC11851252

[26] Ye D Zhang Y Zhao X Zhou H Guo J Yang M . MRGPRX2 gain-of-function mutation drives enhanced mast cell reactivity in chronic spontaneous urticaria. J Allergy Clin Immunol. (2025) 156:159–70. doi: 10.1016/j.jaci.2025.03.007. PMID: 40113019

[27] Sutaria N Adawi W Goldberg R Roh YS Choi J Kwatra SG . Itch: pathogenesis and treatment. J Am Acad Dermatol. (2022) 86:17–34. doi: 10.1016/j.jaad.2021.07.078. PMID: 34648873

[28] Andersen HH Akiyama T Nattkemper LA van Laarhoven A Elberling J Yosipovitch G . Alloknesis and hyperknesis-mechanisms, assessment methodology, and clinical implications of itch sensitization. Pain. (2018) 159:1185–97. doi: 10.1097/j.pain.0000000000001220. PMID: 29659469

[29] Singto T Filor V Vidak J Klopfleisch R Bäumer W . Dendritic cells under allergic condition enhance the activation of pruritogen-responsive neurons via inducing itch receptors in a co-culture study. BMC Immunol. (2024) 25:17. doi: 10.1186/s12865-024-00604-4. PMID: 38347451 PMC10863282

[30] Ishiuji Y Coghill RC Patel TS Oshiro Y Kraft RA Yosipovitch G . Distinct patterns of brain activity evoked by histamine-induced itch reveal an association with itch intensity and disease severity in atopic dermatitis. Br J Dermatol. (2009) 161:1072–80. doi: 10.1111/j.1365-2133.2009.09308.x. PMID: 19663870 PMC2784001

[31] Jakobsson JET Ma H Lagerström MC . Neuropeptide Y in itch regulation. Neuropeptides. (2019) 78:101976. doi: 10.1016/j.npep.2019.101976. PMID: 31668651

[32] Zhao Y Yang H Yu S Li Y Zhang L . Pruritus and anxiety symptoms in chronic spontaneous urticaria are associated with altered functional connectivity of the brain. J Neuroradiol. (2025) 52:101355. doi: 10.1016/j.neurad.2025.101355. PMID: 40451461

[33] Garcovich S Maurelli M Gisondi P Peris K Yosipovitch G Girolomoni G . Pruritus as a distinctive feature of type 2 inflammation. Vaccines (Basel). (2021) 9:303. doi: 10.3390/vaccines9030303. PMID: 33807098 PMC8005108

[34] Trier AM Ver Heul AM Fredman A Le V Wang Z Auyeung K . IL-33 potentiates histaminergic itch. J Allergy Clin Immunol. (2024) 153:852–59.e3. doi: 10.1016/j.jaci.2023.08.038. PMID: 37984799 PMC10939899

[35] Hernanz A Muelas G Borbujo J . Plasma neuropeptide pattern in acute idiopathic urticaria. Int Arch Allergy Appl Immunol. (1989) 90:198–200. doi: 10.1159/000235024. PMID: 2573576

[36] Criado PR Criado RFJ Takakura CFH Pagliari C Sotto MN Vasconcellos C . Immunoelectron microscopy study of superficial skin nerves in drug-induced acute urticaria. Bras Dermatol. (2012) 87:375–81. doi: 10.1590/s0365-05962012000300004. PMID: 22714751

[37] Lang DM Sheikh J Joshi S Bernstein JA . Endotypes, phenotypes, and biomarkers in chronic spontaneous urticaria: Evolving toward personalized medicine. Ann Allergy Asthma Immunol. (2025) 134:408–17.e3. doi: 10.1016/j.anai.2024.10.026. PMID: 39490777

[38] Larenas-Linnemann D . Biomarkers of autoimmune chronic spontaneous urticaria. Curr Allergy Asthma Rep. (2023) 23:655–64. doi: 10.1007/s11882-023-01117-7. PMID: 38064133

[39] Gaudenzio N Sibilano R Marichal T Starkl P Reber LL Cenac N . Different activation signals induce distinct mast cell degranulation strategies. J Clin Invest. (2016) 126:3981–98. doi: 10.1172/JCI85538. PMID: 27643442 PMC5096814

[40] Su Küçük Ö Yücel MB . Clinical and molecular aspects of managing chronic spontaneous urticaria: Identifying endotypes, phenotypes, and determinants of treatment response and resistance. Front Allergy. (2025) 6:1706705. doi: 10.3389/falgy.2025.1706705. PMID: 41608596 PMC12834803

[41] Choi JE Di Nardo A . Skin neurogenic inflammation. Semin Immunopathol. (2018) 40:249–59. doi: 10.1007/s00281-018-0675-z. PMID: 29713744 PMC6047518

[42] Kim HB Um JY Chung BY Park CW Kim HO . Advances in pathophysiology and therapeutic paradigm shifts in chronic spontaneous urticaria: A narrative review. Dermatol Ther (Heidelb). (2026) 16(2):763–88. doi: 10.1007/s13555-025-01600-1. PMID: 41410910 PMC12936329

[43] Mendoza RP Anderson CC Fudge DH Roede JR Brown JM . Metabolic consequences of IgE- and non-IgE-mediated mast cell degranulation. J Immunol. (2021) 207:2637–48. doi: 10.4049/jimmunol.2001278. PMID: 34732470 PMC8612977

[44] Lee R Bernstein JA . Chronic spontaneous urticaria and chronic inducible urticaria. J Allergy Clin Immunol. (2025) 156:546–56. doi: 10.1016/j.jaci.2025.05.019. PMID: 40451490

[45] Bizjak M Košnik M . Key differences between chronic inducible and spontaneous urticaria. Front Allergy. (2024) 5:1487831. doi: 10.3389/falgy.2024.1487831. PMID: 39483682 PMC11524999

[46] Terhorst-Molawi D Hawro T Grekowitz E Kiefer L Merchant K Alvarado D . Anti-KIT antibody, barzolvolimab, reduces skin mast cells and disease activity in chronic inducible urticaria. Allergy. (2023) 78:1269–79. doi: 10.1111/all.15585. PMID: 36385701

[47] Murphy GM Zollman PE Greaves MW Winkelmann RK . Symptomatic dermographism (factitious urticaria)--passive transfer experiments from human to monkey. Br J Dermatol. (1987) 116:801–4. doi: 10.1111/j.1365-2133.1987.tb04898.x. PMID: 3620341

[48] McNeil BD Pundir P Meeker S Han L Undem BJ Kulka M . Identification of a mast-cell-specific receptor crucial for pseudo-allergic drug reactions. Nature. (2015) 519:237–41. doi: 10.1038/nature14022. PMID: 25517090 PMC4359082

[49] Li F Yang W Jiang H Guo C Huang AJW Hu H . TRPV1 activity and substance P release are required for corneal cold nociception. Nat Commun. (2019) 10:5678. doi: 10.1038/s41467-019-13536-0. PMID: 31831729 PMC6908618

[50] Zheng W Wang J Zhu W Xu C He S . Upregulated expression of substance P in basophils of the patients with chronic spontaneous urticaria: induction of histamine release and basophil accumulation by substance P. Cell Biol Toxicol. (2016) 32:217–28. doi: 10.1007/s10565-016-9330-4. PMID: 27147256

[51] Metz M Krull C Hawro T Saluja R Groffik A Stanger C . Substance P is upregulated in the serum of patients with chronic spontaneous urticaria. J Invest Dermatol. (2014) 134:2833–6. doi: 10.1038/jid.2014.226. PMID: 24844859

[52] Fadaee J Khoshkhui M Emadzadeh M Hashemy SI Farid Hosseini R Jabbari Azad F . Evaluation of serum substance P level in chronic urticaria and correlation with disease severity. Iran J Allergy Asthma Immunol. (2020) 19:18–26. doi: 10.18502/ijaai.v19i1.2414. PMID: 32245317

[53] Basak PY Erturan I Yuksel O Kazanoglu OO Vural H . Evaluation of serum neuropeptide levels in patients with chronic urticaria. Indian J Dermatol Venereol Leprol. (2014) 80:483. doi: 10.4103/0378-6323.140345. PMID: 25201865

[54] Vena GA Cassano N Di Leo E Calogiuri GF Nettis E . Focus on the role of substance P in chronic urticaria. Clin Mol Allergy. (2018) 16:24. doi: 10.1186/s12948-018-0101-z. PMID: 30473632 PMC6240950

[55] Tedeschi A Lorini M Asero R . No evidence of increased serum substance P levels in chronic urticaria patients with and without demonstrable circulating vasoactive factors. Clin Exp Dermatol. (2005) 30:171–5. doi: 10.1111/j.1365-2230.2005.01732.x. PMID: 15725248

[56] Cima K Vogelsinger H Kähler CM . Sensory neuropeptides are potent chemoattractants for human basophils *in vitro*. Regul Pept. (2010) 160:42–8. doi: 10.1016/j.regpep.2009.12.013. PMID: 20035805

[57] West PW Chéret J Bahri R Kiss O Wu Z Macphee CH . The MRGPRX2-substance P pathway regulates mast cell migration. iScience. (2024) 27:110984. doi: 10.1016/j.isci.2024.110984. PMID: 39435146 PMC11492034

[58] Borici-Mazi R Kouridakis S Kontou-Fili K . Cutaneous responses to substance P and calcitonin gene-related peptide in chronic urticaria: the effect of cetirizine and dimethindene. Allergy. (1999) 54:46–56. doi: 10.1034/j.1398-9995.1999.00726.x. PMID: 10195357

[59] Le Greves P Nyberg F Terenius L Hökfelt T . Calcitonin gene-related peptide is a potent inhibitor of substance P degradation. Eur J Pharmacol. (1985) 115:309–11. doi: 10.1016/0014-2999(85)90706-x. PMID: 2415371

[60] Boyvadoglu C Ulusal H Taysı S Ozaydin-Yavuz G Yavuz IH Korkmaz P I . Effects of omalizumab on serum levels of substance P, calcitonin gene-related peptide, neuropeptide Y, and interleukin-31 in patients with chronic spontaneous urticaria. Mediators Inflammation. (2023) 2023:8087274. doi: 10.1155/2023/8087274. PMID: 37795408 PMC10547569

[61] Başak PY Vural H Kazanoglu OO Erturan I Buyukbayram HI . Effects of loratadine and cetirizine on serum levels of neuropeptides in patients with chronic urticaria. Int J Dermatol. (2014) 53:1526–30. doi: 10.1111/ijd.12590. PMID: 25209952

[62] Kay AB Ying S Ardelean E Mlynek A Kita H Clark P . Calcitonin gene-related peptide and vascular endothelial growth factor are expressed in lesional but not uninvolved skin in chronic spontaneous urticaria. Clin Exp Allergy. (2014) 44:1053–60. doi: 10.1111/cea.12348. PMID: 24902612

[63] Levite M . Neurotransmitters activate T-cells and elicit crucial functions via neurotransmitter receptors. Curr Opin Pharmacol. (2008) 8:460–71. doi: 10.1016/j.coph.2008.05.001. PMID: 18579442

[64] Kwiatkowska D Reich A . Role of mast cells in the pathogenesis of pruritus in mastocytosis. Acta Derm Venereol. (2021) 101:adv00583. doi: 10.2340/actadv.v101.350. PMID: 34642766 PMC9425624

[65] Assas BM Pennock JI Miyan JA . Calcitonin gene-related peptide is a key neurotransmitter in the neuro-immune axis. Front Neurosci. (2014) 8:23. doi: 10.3389/fnins.2014.00023. PMID: 24592205 PMC3924554

[66] Rössing K Novak N Mommert S Pfab F Gehring M Wedi B . Brain-derived neurotrophic factor is increased in serum and skin levels of patients with chronic spontaneous urticaria. Clin Exp Allergy. (2011) 41:1392–9. doi: 10.1111/j.1365-2222.2011.03795.x. PMID: 21676041

[67] Bonini S Lambiase A Bonini S Angelucci F Magrini L Manni L . Circulating nerve growth factor levels are increased in humans with allergic diseases and asthma. Proc Natl Acad Sci USA. (1996) 93:10955–60. doi: 10.1073/pnas.93.20.10955. PMID: 8855290 PMC38265

[68] Ozseker F Büyüköztürk S Gelincik A Depboylu B Genç S Giriş M . Neurotrophins: are they meaningful in chronic spontaneous urticaria? Asian Pac J Allergy Immunol. (2008) 26:83–8. 19054925

[69] Kulumbegov B Chikovani T Gotua M Kikodze N Magen E . Interleukin-33, endothelin-1, and inflammatory parameters in chronic spontaneous urticaria. Allergy Asthma Proc. (2023) 44:429–35. doi: 10.2500/aap.2023.44.230051. PMID: 37919851

[70] Kühn H Kolkhir P Babina M Düll M Frischbutter S Fok JS . Mas-related G protein-coupled receptor X2 and its activators in dermatologic allergies. J Allergy Clin Immunol. (2021) 147:456–69. doi: 10.1016/j.jaci.2020.08.027. PMID: 33071069

[71] Smith CH Atkinson B Morris RW Hayes N Foreman JC Lee TH . Cutaneous responses to vasoactive intestinal polypeptide in chronic idiopathic urticaria. Lancet. (1992) 339:91–3. doi: 10.1016/0140-6736(92)91000-x. PMID: 1370236

[72] Ruppenstein A Limberg MM Loser K Kremer AE Homey B Raap U . Involvement of neuro-immune interactions in pruritus with special focus on receptor expressions. Front Med (Lausanne). (2021) 8:627985. doi: 10.3389/fmed.2021.627985. PMID: 33681256 PMC7930738

[73] Fujisawa D Kashiwakura J Kita H Kikukawa Y Fujitani Y Sasaki-Sakamoto T . Expression of Mas-related gene X2 on mast cells is upregulated in the skin of patients with severe chronic urticaria. J Allergy Clin Immunol. (2014) 134:622–33.e9. doi: 10.1016/j.jaci.2014.05.004. PMID: 24954276

[74] Cao TBT Cha HY Yang EM Ye YM . Elevated MRGPRX2 levels related to disease severity in patients with chronic spontaneous urticaria. Allergy Asthma Immunol Res. (2021) 13:498–506. doi: 10.4168/aair.2021.13.3.498. PMID: 33733642 PMC7984951

[75] Ding Y Dang B Wang Y Zhao C An H . Artemisinic acid attenuated symptoms of substance P-induced chronic urticaria in a mice model and mast cell degranulation via Lyn/PLC-p38 signal pathway. Int Immunopharmacol. (2022) 113:109437. doi: 10.1016/j.intimp.2022.109437. PMID: 36403523

[76] Ding Y Dang B Zhang Y Hu S Wang Y Zhao C . Paeonol attenuates substance P-induced urticaria by inhibiting Src kinase phosphorylation in mast cells. Cell Immunol. (2023) 388-389:104728. doi: 10.1016/j.cellimm.2023.104728. PMID: 37224634

[77] Russell FA King R Smillie SJ Kodji X Brain SD . Calcitonin gene-related peptide: physiology and pathophysiology. Physiol Rev. (2014) 94:1099–142. doi: 10.1152/physrev.00034.2013. PMID: 25287861 PMC4187032

[78] Dunzendorfer S Meierhofer C Wiedermann CJ . Signaling in neuropeptide-induced migration of human eosinophils. J Leukoc Biol. (1998) 64:828–34. doi: 10.1002/jlb.64.6.828. PMID: 9850167

[79] Bienenstock J Tomioka M Matsuda H Stead RH Quinonez G Simon GT . The role of mast cells in inflammatory processes: evidence for nerve/mast cell interactions. Int Arch Allergy Appl Immunol. (1987) 82:238–43. doi: 10.1159/000234197. PMID: 2437039

[80] Lee J Yamamoto T Hayashi S Kuramoto H Kadowaki M . Enhancement of CGRP sensory afferent innervation in the gut during the development of food allergy in an experimental murine model. Biochem Biophys Res Commun. (2013) 430:895–900. doi: 10.1016/j.bbrc.2012.12.058. PMID: 23261435

[81] Kaplan DR Miller FD . Neurotrophin signal transduction in the nervous system. Curr Opin Neurobiol. (2000) 10:381–91. doi: 10.1016/s0959-4388(00)00092-1. PMID: 10851172

[82] Rocco ML Soligo M Manni L Aloe L . Nerve growth factor: early studies and recent clinical trials. Curr Neuropharmacol. (2018) 16:1455–65. doi: 10.2174/1570159X16666180412092859. PMID: 29651949 PMC6295934

[83] Raap U Goltz C Deneka N Bruder M Renz H Kapp A . Brain-derived neurotrophic factor is increased in atopic dermatitis and modulates eosinophil functions compared with that seen in nonatopic subjects. J Allergy Clin Immunol. (2005) 115:1268–75. doi: 10.1016/j.jaci.2005.02.007. PMID: 15940146

[84] Jiao Q Lohse K Rauber-Ellinghaus MM Kolkhir P Frischbutter S Scheffel J . Basophil and eosinophil recruitment to skin lesions is linked to features of autoimmune chronic spontaneous urticaria including basopenia. J Eur Acad Dermatol Venereol. (2025) 39:e155–7. doi: 10.1111/jdv.20204. PMID: 38923599

[85] Manils J Webb LV Howes A Janzen J Boeing S Bowcock AM . CARD14E138A signalling in keratinocytes induces TNF-dependent skin and systemic inflammation. Elife. (2020) 9:e56720. doi: 10.7554/eLife.56720. PMID: 32597759 PMC7351492

[86] Marconi A Terracina M Fila C Franchi J Bonté F Romagnoli G . Expression and function of neurotrophins and their receptors in cultured human keratinocytes. J Invest Dermatol. (2003) 121:1515–21. doi: 10.1111/j.1523-1747.2003.12624.x. PMID: 14675204

[87] Sharma P Sharma PK Chitkara A Rani S . To evaluate the role and relevance of cytokines IL-17, IL-18, IL-23 and TNF-α and their correlation with disease severity in chronic urticaria. Indian Dermatol Online J. (2020) 11:594–7. doi: 10.4103/idoj.IDOJ_396_19. PMID: 32832449 PMC7413447

[88] Hermes B Zuberbier T Haas N Henz BM . Decreased cutaneous expression of stem cell factor and of the p75NGF receptor in urticaria. Br J Dermatol. (2003) 148:411–7. doi: 10.1046/j.1365-2133.2003.05167.x. PMID: 12653731

[89] Magnúsdóttir EI Grujic M Bergman J Pejler G Lagerström MC . Mouse connective tissue mast cell proteases tryptase and carboxypeptidase A3 play protective roles in itch induced by endothelin-1. J Neuroinflamm. (2020) 17:123. doi: 10.1186/s12974-020-01795-4. PMID: 32321525 PMC7175568

[90] Metz M Schäfer B Tsai M Maurer M Galli SJ . Evidence that the endothelin A receptor can enhance IgE-dependent anaphylaxis in mice. J Allergy Clin Immunol. (2011) 128:424–6.e1. doi: 10.1016/j.jaci.2011.04.012. PMID: 21555149 PMC3565840

[91] Kulka M Sheen CH Tancowny BP Grammer LC Schleimer RP . Neuropeptides activate human mast cell degranulation and chemokine production. Immunology. (2008) 123:398–410. doi: 10.1111/j.1365-2567.2007.02705.x. PMID: 17922833 PMC2433325

[92] Subramanian H Gupta K Ali H . Roles of Mas-related G protein-coupled receptor X2 on mast cell-mediated host defense, pseudoallergic drug reactions, and chronic inflammatory diseases. J Allergy Clin Immunol. (2016) 138:700–10. doi: 10.1016/j.jaci.2016.04.051. PMID: 27448446 PMC5014572

[93] Tsou AM Yano H Parkhurst CN Mahlakõiv T Chu C Zhang W . Neuropeptide regulation of non-redundant ILC2 responses at barrier surfaces. Nature. (2022) 611:787–93. doi: 10.1038/s41586-022-05297-6. PMID: 36323781 PMC10225046

[94] Wallrapp A Riesenfeld SJ Burkett PR Abdulnour RE Nyman J Dionne D . The neuropeptide NMU amplifies ILC2-driven allergic lung inflammation. Nature. (2017) 549:351–6. doi: 10.1038/nature24029. PMID: 28902842 PMC5746044

[95] Xu JF Liu L Liu Y Lu KX Zhang J Zhu YJ . Spinal Nmur2-positive neurons play a crucial role in mechanical itch. J Pain. (2024) 25:104504. doi: 10.1016/j.jpain.2024.02.018. PMID: 38442838

[96] Liu XY Wan L Huo FQ Barry DM Li H Zhao ZQ . B-type natriuretic peptide is neither itch-specific nor functions upstream of the GRP-GRPR signaling pathway. Mol Pain. (2014) 10:4. doi: 10.1186/1744-8069-10-4. PMID: 24438367 PMC3930899

[97] Tamari M Ver Heul AM Kim BS . Immunosensation: neuroimmune cross talk in the skin. Annu Rev Immunol. (2021) 39:369–93. doi: 10.1146/annurev-immunol-101719-113805. PMID: 33561366

[98] Steinhoff M Ahmad F Pandey A Datsi A AlHammadi A Al-Khawaga S . Neuroimmune communication regulating pruritus in atopic dermatitis. J Allergy Clin Immunol. (2022) 149:1875–98. doi: 10.1016/j.jaci.2022.03.010. PMID: 35337846

[99] Haas N Toppe E Henz BM . Microscopic morphology of different types of urticaria. Arch Dermatol. (1998) 134:41–6. doi: 10.1001/archderm.134.1.41. PMID: 9449908

[100] Johansson O Wang L Hilliges M Liang Y . Intraepidermal nerves in human skin: PGP 9.5 immunohistochemistry with special reference to the nerve density in skin from different body regions. J Peripher Nerv Syst. (1999) 4:43–52. 10197064

[101] Marek-Jozefowicz L Nedoszytko B Grochocka M Żmijewski MA Czajkowski R Cubała WJ . Molecular mechanisms of neurogenic inflammation of the skin. Int J Mol Sci. (2023) 24:5001. doi: 10.3390/ijms24055001. PMID: 36902434 PMC10003326

[102] Kruger L Silverman JD Mantyh PW Sternini C Brecha NC . Peripheral patterns of calcitonin-gene-related peptide general somatic sensory innervation: cutaneous and deep terminations. J Comp Neurol. (1989) 280:291–302. doi: 10.1002/cne.902800210. PMID: 2784448

[103] Kunisada T Lu SZ Yoshida H Nishikawa S Nishikawa S Mizoguchi M . Murine cutaneous mastocytosis and epidermal melanocytosis induced by keratinocyte expression of transgenic stem cell factor. J Exp Med. (1998) 187:1565–73. doi: 10.1084/jem.187.10.1565. PMID: 9584135 PMC2212288

[104] Ye J Lai Y . Keratinocytes: new perspectives in inflammatory skin diseases. Trends Mol Med. (2025) 31:1103–13. doi: 10.1016/j.molmed.2025.03.012. PMID: 40246604

[105] Komi DEA Khomtchouk K Santa Maria PL . A review of the contribution of mast cells in wound healing: involved molecular and cellular mechanisms. Clin Rev Allergy Immunol. (2020) 58:298–312. doi: 10.1007/s12016-019-08729-w. PMID: 30729428

[106] Franke K Kirchner M Mertins P Zuberbier T Babina M . The SCF/KIT axis in human mast cells: Capicua acts as potent KIT repressor and ERK predominates PI3K. Allergy. (2022) 77:3337–49. doi: 10.1111/all.15396. PMID: 35652819

[107] Ho CCM Chhabra A Starkl P Schnorr PJ Wilmes S Moraga I . Decoupling the functional pleiotropy of stem cell factor by tuning c-Kit signaling. Cell. (2017) 168:1041–52.e18. doi: 10.1016/j.cell.2017.02.011. PMID: 28283060 PMC5526607

[108] Smrž D Bandara G Beaven MA Metcalfe DD Gilfillan AM . Prevention of F-actin assembly switches the response to SCF from chemotaxis to degranulation in human mast cells. Eur J Immunol. (2013) 43:1873–82. doi: 10.1002/eji.201243214. PMID: 23616175 PMC3798040

[109] Furuno T Ito A Koma Y Watabe K Yokozaki H Bienenstock J . The spermatogenic Ig superfamily/synaptic cell adhesion molecule mast-cell adhesion molecule promotes interaction with nerves. J Immunol. (2005) 174:6934–42. doi: 10.4049/jimmunol.174.11.6934. PMID: 15905536

[110] Ito A Oonuma J . Direct interaction between nerves and mast cells mediated by the SgIGSF/SynCAM adhesion molecule. J Pharmacol Sci. (2006) 102:1–5. doi: 10.1254/jphs.cpj06014x. PMID: 16936456

[111] Magadmi R Meszaros J Damanhouri ZA Seward EP . Secretion of mast cell inflammatory mediators is enhanced by CADM1-dependent adhesion to sensory neurons. Front Cell Neurosci. (2019) 13:262. doi: 10.3389/fncel.2019.00262. PMID: 31275114 PMC6591473

[112] Siiskonen H Harvima I . Mast cells and sensory nerves contribute to neurogenic inflammation and pruritus in chronic skin inflammation. Front Cell Neurosci. (2019) 13:422. doi: 10.3389/fncel.2019.00422. PMID: 31619965 PMC6759746

[113] Furuno T Hagiyama M Sekimura M Okamoto K Suzuki R Ito A . Cell adhesion molecule 1 (CADM1) on mast cells promotes interaction with dorsal root ganglion neurites by heterophilic binding to nectin-3. J Neuroimmunol. (2012) 250:50–8. doi: 10.1016/j.jneuroim.2012.05.016. PMID: 22703826

[114] Hagiyama M Inoue T Furuno T Iino T Itami S Nakanishi M . Increased expression of cell adhesion molecule 1 by mast cells as a cause of enhanced nerve-mast cell interaction in a hapten-induced mouse model of atopic dermatitis. Br J Dermatol. (2013) 168:771–8. doi: 10.1111/bjd.12108. PMID: 23106683

[115] Yuki A Ansai O Abe R . CADM1 expression of mast cells in mycosis fungoides. J Am Acad Dermatol. (2020) 82:e143–4. doi: 10.1016/j.jaad.2019.12.017. PMID: 31857111

[116] Church MK Kolkhir P Metz M Maurer M . The role and relevance of mast cells in urticaria. Immunol Rev. (2018) 282:232–47. doi: 10.1111/imr.12632. PMID: 29431202

[117] Irani AA Schechter NM Craig SS DeBlois G Schwartz LB . Two types of human mast cells that have distinct neutral protease compositions. Proc Natl Acad Sci USA. (1986) 83:4464–8. doi: 10.1073/pnas.83.12.4464. PMID: 3520574 PMC323754

[118] Smith CH Kepley C Schwartz LB Lee TH . Mast cell number and phenotype in chronic idiopathic urticaria. J Allergy Clin Immunol. (1995) 96:360–4. doi: 10.1016/s0091-6749(95)70055-2. PMID: 7560638

[119] Molfetta R Carnevale A Marangio C Putro E Paolini R . Beyond the “Master” role in allergy: Insights into intestinal mast cell plasticity and gastrointestinal diseases. Biomedicines. (2025) 13:320. doi: 10.3390/biomedicines13020320. PMID: 40002733 PMC11853218

[120] Zhang Z Ernst PB Kiyono H Kurashima Y . Utilizing mast cells in a positive manner to overcome inflammatory and allergic diseases. Front Immunol. (2022) 13:937120. doi: 10.3389/fimmu.2022.937120. PMID: 36189267 PMC9518231

[121] Li Q Zhao C . An *in vitro* study on substance P-stimulated neuro-immune mechanism of mast cell degranulation. Lin Chuang Er Bi Yan Hou Tou Jing Wai Ke Za Zhi. (2015) 29:1118–20. 26514006

[122] Steinhoff M Buddenkotte J Lerner EA . Role of mast cells and basophils in pruritus. Immunol Rev. (2018) 282:248–64. doi: 10.1111/imr.12635. PMID: 29431207

[123] Schnakenberg M Thomas C Schmelz M Rukwied R . Nerve growth factor sensitizes nociceptors to C-fibre selective supra-threshold electrical stimuli in human skin. Eur J Pain. (2021) 25:385–97. doi: 10.1002/ejp.1678. PMID: 33064901

[124] Cevikbas F Wang X Akiyama T Kempkes C Savinko T Antal A . A sensory neuron-expressed IL-31 receptor mediates T helper cell-dependent itch: Involvement of TRPV1 and TRPA1. J Allergy Clin Immunol. (2014) 133:448–60. doi: 10.1016/j.jaci.2013.10.048. PMID: 24373353 PMC3960328

[125] Oetjen LK Mack MR Feng J Whelan TM Niu H Guo CJ . Sensory neurons co-opt classical immune signaling pathways to mediate chronic itch. Cell. (2017) 171:217–28.e13. doi: 10.1016/j.cell.2017.08.006. PMID: 28890086 PMC5658016

[126] van Houwelingen AH Kool M de Jager SC Redegeld FA van Heuven-Nolsen D Kraneveld AD . Mast cell-derived TNF-alpha primes sensory nerve endings in a pulmonary hypersensitivity reaction. J Immunol. (2002) 168:5297–302. doi: 10.4049/jimmunol.168.10.5297. PMID: 11994487

[127] Lau AH Chow SS Ng YS . Immunologically induced histamine release from rat peritoneal mast cells is enhanced by low levels of substance P. Eur J Pharmacol. (2001) 414:295–303. doi: 10.1016/s0014-2999(01)00805-6. PMID: 11239931

[128] van der Kleij HPM Ma D Redegeld FAM Kraneveld AD Nijkamp FP Bienenstock J . Functional expression of neurokinin 1 receptors on mast cells induced by IL-4 and stem cell factor. J Immunol. (2003) 171:2074–9. doi: 10.4049/jimmunol.171.4.2074. PMID: 12902513

[129] Green DP Limjunyawong N Gour N Pundir P Dong X . A mast-cell-specific receptor mediates neurogenic inflammation and pain. Neuron. (2019) 101:412–20.e3. doi: 10.1016/j.neuron.2019.01.012. PMID: 30686732 PMC6462816

[130] Nagamine M Kaitani A Izawa K Ando T Yoshikawa A Nakamura M . Neuronal substance P-driven MRGPRX2-dependent mast cell degranulation products differentially promote vascular permeability. Front Immunol. (2024) 15:1477072. doi: 10.3389/fimmu.2024.1477072. PMID: 39640264 PMC11617324

[131] He X Yang X Qin L Zhang Q Ji X Tang W . Amphotericin B for injection triggers degranulation of human LAD2 mast cells by MRGPRX2 and pseudo-allergic reactions in mice via MRGPRB2 activation. Immunol Res. (2024) 72:1337–49. doi: 10.1007/s12026-024-09532-2. PMID: 39223434

[132] Erickson S Heul AV Kim BS . New and emerging treatments for inflammatory itch. Ann Allergy Asthma Immunol. (2021) 126:13–20. doi: 10.1016/j.anai.2020.05.028. PMID: 32497711

[133] Rychter JW Van Nassauw L Timmermans JP Akkermans LMA Westerink RHS Kroese ABA . CGRP1 receptor activation induces piecemeal release of protease-1 from mouse bone marrow-derived mucosal mast cells. Neurogastroenterol Motil. (2011) 23:e57–68. doi: 10.1111/j.1365-2982.2010.01617.x. PMID: 20964790

[134] McDougall JJ Barin AK . The role of joint nerves and mast cells in the alteration of vasoactive intestinal peptide (VIP) sensitivity during inflammation progression in rats. Br J Pharmacol. (2005) 145:104–13. doi: 10.1038/sj.bjp.0706169. PMID: 15723091 PMC1576122

[135] Verma AK Manohar M Upparahalli Venkateshaiah S Mishra A . Neuroendocrine cells derived chemokine vasoactive intestinal polypeptide (VIP) in allergic diseases. Cytokine Growth Factor Rev. (2017) 38:37–48. doi: 10.1016/j.cytogfr.2017.09.002. PMID: 28964637 PMC5705463

[136] McCary C Tancowny BP Catalli A Grammer LC Harris KE Schleimer RP . Substance P downregulates expression of the high affinity IgE receptor (FcepsilonRI) by human mast cells. J Neuroimmunol. (2010) 220:17–24. doi: 10.1016/j.jneuroim.2009.12.006. PMID: 20117843 PMC3712287

[137] Zschiebsch K Fischer C Wilken-Schmitz A Geisslinger G Channon K Watschinger K . Mast cell tetrahydrobiopterin contributes to itch in mice. J Cell Mol Med. (2019) 23:985–1000. doi: 10.1111/jcmm.13999. PMID: 30450838 PMC6349351

[138] Kabata H Artis D . Neuro-immune crosstalk and allergic inflammation. J Clin Invest. (2019) 129:1475–82. doi: 10.1172/JCI124609. PMID: 30829650 PMC6436850

[139] Drake MG Lebold KM Roth-Carter QR Pincus AB Blum ED Proskocil BJ . Eosinophil and airway nerve interactions in asthma. J Leukoc Biol. (2018) 104:61–7. doi: 10.1002/JLB.3MR1117-426R. PMID: 29633324 PMC6541210

[140] Lorenzo GD Mansueto P Melluso M Candore G Cigna D Pellitteri ME . Blood eosinophils and serum eosinophil cationic protein in patients with acute and chronic urticaria. Mediators Inflammation. (1996) 5:113–5. doi: 10.1155/S0962935196000191. PMID: 18475708 PMC2365782

[141] Saleh AA Al-Obaidi AM Behiry EG Hamed AM . Serum levels of eosinophil-derived neurotoxin in patients with chronic urticaria. J Clin Aesthet Dermatol. (2020) 13:21–3. PMC7577334 33133337

[142] Toyoda M Maruyama T Morohashi M Bhawan J . Free eosinophil granules in urticaria: a correlation with the duration of wheals. Am J Dermatopathol. (1996) 18:49–57. doi: 10.1097/00000372-199602000-00008. PMID: 8721591

[143] Lee JJ Protheroe CA Luo H Ochkur SI Scott GD Zellner KR . Eosinophil-dependent skin innervation and itching following contact toxicant exposure in mice. J Allergy Clin Immunol. (2015) 135:477–87. doi: 10.1016/j.jaci.2014.07.003. PMID: 25129680 PMC4464693

[144] Nakashima C Ishida Y Kitoh A Otsuka A Kabashima K . Interaction of peripheral nerves and mast cells, eosinophils, and basophils in the development of pruritus. Exp Dermatol. (2019) 28:1405–11. doi: 10.1111/exd.14014. PMID: 31365150

[145] Wang F Trier AM Li F Kim S Chen Z Chai JN . A basophil-neuronal axis promotes itch. Cell. (2021) 184:422–40.e17. doi: 10.1016/j.cell.2020.12.033. PMID: 33450207 PMC7878015

[146] Huang AH Chichester KL Saini SS . Association of basophil parameters with disease severity and duration in chronic spontaneous urticaria (CSU). J Allergy Clin Immunol Pract. (2020) 8:793–5.e6. doi: 10.1016/j.jaip.2019.08.004. PMID: 31421279 PMC7012729

[147] Bartko EA Elberling J Blom LH Poulsen LK Jensen BM . Elevated, FcϵRI-dependent MRGPRX2 expression on basophils in chronic urticaria. Skin Health Dis. (2023) 3:e195. doi: 10.1002/ski2.195. PMID: 37275407 PMC10233071

[148] Takahashi S Ochiai S Jin J Takahashi N Toshima S Ishigame H . Sensory neuronal STAT3 is critical for IL-31 receptor expression and inflammatory itch. Cell Rep. (2023) 42:113433. doi: 10.1016/j.celrep.2023.113433. PMID: 38029739

[149] Datsi A Steinhoff M Ahmad F Alam M Buddenkotte J . Interleukin-31: The “itchy” cytokine in inflammation and therapy. Allergy. (2021) 76:2982–97. doi: 10.1111/all.14791. PMID: 33629401

[150] Raap U Gehring M Kleiner S Rüdrich U Eiz-Vesper B Haas H . Human basophils are a source of - and are differentially activated by - IL-31. Clin Exp Allergy. (2017) 47:499–508. doi: 10.1111/cea.12875. PMID: 28000952

[151] Altrichter S Hawro T Hänel K Czaja K Lüscher B Maurer M . Successful omalizumab treatment in chronic spontaneous urticaria is associated with lowering of serum IL-31 levels. J Eur Acad Dermatol Venereol. (2016) 30:454–5. doi: 10.1111/jdv.12831. PMID: 25371135

[152] Karstarli Bakay OS Demir B Cicek D Erol D Aşçı Toraman Z Gural Y . In chronic spontaneous urticaria, IgE and C-reactive protein are linked to distinct microRNAs and interleukin-31. Clin Transl Allergy. (2023) 13:e12290. doi: 10.1002/clt2.12290. PMID: 37632245 PMC10405150

[153] Cardoso V Chesné J Ribeiro H García-Cassani B Carvalho T Bouchery T . Neuronal regulation of type 2 innate lymphoid cells via neuromedin U. Nature. (2017) 549:277–81. doi: 10.1038/nature23469. PMID: 28869974 PMC5714273

[154] Nagashima H Mahlakõiv T Shih HY Davis FP Meylan F Huang Y . Neuropeptide CGRP limits group 2 innate lymphoid cell responses and constrains type 2 inflammation. Immunity. (2019) 51:682–95.e6. doi: 10.1016/j.immuni.2019.06.009. PMID: 31353223 PMC6801073

[155] Qin M Fang Y Zheng Q Peng M Wang L Sang X . Tissue microenvironment induces tissue specificity of ILC2. Cell Death Discov. (2024) 10:324. doi: 10.1038/s41420-024-02096-y. PMID: 39013890 PMC11252336

[156] Bernink JH Ohne Y Teunissen MBM Wang J Wu J Krabbendam L . c-Kit-positive ILC2s exhibit an ILC3-like signature that may contribute to IL-17-mediated pathologies. Nat Immunol. (2019) 20:992–1003. doi: 10.1038/s41590-019-0423-0. PMID: 31263279

[157] Mijouin L Hillion M Ramdani Y Jaouen T Duclairoir-Poc C Follet-Gueye ML . Effects of a skin neuropeptide (substance p) on cutaneous microflora. PloS One. (2013) 8:e78773. doi: 10.1371/journal.pone.0078773. PMID: 24250813 PMC3826737

[158] Shi X Wang L Clark JD Kingery WS . Keratinocytes express cytokines and nerve growth factor in response to neuropeptide activation of the ERK1/2 and JNK MAPK transcription pathways. Regul Pept. (2013) 186:92–103. doi: 10.1016/j.regpep.2013.08.001. PMID: 23958840 PMC3799830

[159] Dallos A Kiss M Polyánka H Dobozy A Kemény L Husz S . Effects of the neuropeptides substance P, calcitonin gene-related peptide, vasoactive intestinal polypeptide and galanin on the production of nerve growth factor and inflammatory cytokines in cultured human keratinocytes. Neuropeptides. (2006) 40:251–63. doi: 10.1016/j.npep.2006.06.002. PMID: 16904178

[160] Wilson SR Thé L Batia LM Beattie K Katibah GE McClain SP . The epithelial cell-derived atopic dermatitis cytokine TSLP activates neurons to induce itch. Cell. (2013) 155:285–97. doi: 10.1016/j.cell.2013.08.057. PMID: 24094650 PMC4041105

[161] Kay AB Clark P Maurer M Ying S . Elevations in T-helper-2-initiating cytokines (interleukin-33, interleukin-25 and thymic stromal lymphopoietin) in lesional skin from chronic spontaneous (‘idiopathic’) urticaria. Br J Dermatol. (2015) 172:1294–302. doi: 10.1111/bjd.13621. PMID: 25523947

[162] Dobrican-Băruța CT Deleanu DM Iancu M Muntean IA Nedelea I Bălan RG . Exploring the impact of IL-33 gene polymorphism (rs1929992) on susceptibility to chronic spontaneous urticaria and its association with serum interleukin-33 levels. Int J Mol Sci. (2024) 25:13709. doi: 10.3390/ijms252413709. PMID: 39769469 PMC11677185

[163] Dobrican-Băruța CT Deleanu DM Muntean IA Nedelea I Bălan RG Filip GA . The alarmin triad-IL-25, IL-33, and TSLP-serum levels and their clinical implications in chronic spontaneous urticaria. Int J Mol Sci. (2024) 25:2026. doi: 10.3390/ijms25042026. PMID: 38396704 PMC10889490

[164] Liu B Tai Y Achanta S Kaelberer MM Caceres AI Shao X . IL-33/ST2 signaling excites sensory neurons and mediates itch response in a mouse model of poison ivy contact allergy. Proc Natl Acad Sci USA. (2016) 113:E7572–9. doi: 10.1073/pnas.1606608113. PMID: 27821781 PMC5127381

[165] Kamata Y Tominaga M Takamori K . Mechanisms of itch in atopic dermatitis. Juntendo Med J. (2025) 71:43–50. doi: 10.14789/ejmj.JMJ24-0036-R. PMID: 40109398 PMC11915750

[166] Maurer M Casale TB Saini SS Ben-Shoshan M Giménez-Arnau AM Bernstein JA . Dupilumab in patients with chronic spontaneous urticaria (LIBERTY-CSU CUPID): Two randomized, double-blind, placebo-controlled, phase 3 trials. J Allergy Clin Immunol. (2024) 154:184–94. doi: 10.1016/j.jaci.2024.01.028. PMID: 38431226

[167] Maurer M Casale TB Saini SS Ben-Shoshan M Laws E Maloney J . Dupilumab reduces urticaria activity, itch, and hives in patients with chronic spontaneous urticaria regardless of baseline serum immunoglobulin E levels. Dermatol Ther (Heidelb). (2024) 14:2427–41. doi: 10.1007/s13555-024-01231-y. PMID: 39066978 PMC11393262

[168] LeunigA GianeselliM RussoSJ SwirskiFK . Connection and communication between the nervous and immune systems. Nat Rev Immunol. (2025). doi: 10.1038/s41577-025-01199-6. PMID: 40640567

[169] De A Pal S Singh S Chakroborty D Godse K . An open-label, investigator-initiated, single-centre pilot study to determine the safety and efficacy of tofacitinib in resistant chronic spontaneous urticaria. Indian J Dermatol. (2024) 69:312–6. doi: 10.4103/ijd.ijd_1085_23. PMID: 39296701 PMC11407565

[170] Du N Wang D Yang J . Case report: Exploration of abrocitinib in the treatment of refractory chronic spontaneous urticaria: a case series. Front Immunol. (2024) 15:1466058. doi: 10.3389/fimmu.2024.1466058. PMID: 39469709 PMC11513297

[171] McLaren J Chon Y Gorski KS Bernstein JA Corren J Hayama K . Tezepelumab for the treatment of chronic spontaneous urticaria: Results of the phase 2b INCEPTION study. J Allergy Clin Immunol. (2025) 155:1945–56. doi: 10.1016/j.jaci.2025.01.045. PMID: 39956278

[172] Wollam J Solomon M Villescaz C Lanier M Evans S Bacon C . Inhibition of mast cell degranulation by novel small molecule MRGPRX2 antagonists. J Allergy Clin Immunol. (2024) 154:1033–43. doi: 10.1016/j.jaci.2024.07.002. PMID: 38971540

[173] Al Hamwi G Alnouri MW Verdonck S Leonczak P Chaki S Frischbutter S . Subnanomolar MAS-related G protein-coupled receptor-X2/B2 antagonists with efficacy in human mast cells and disease models. Signal Transduct Target Ther. (2025) 10:128. doi: 10.1038/s41392-025-02209-8. PMID: 40254631 PMC12010006

